# Regulatory mechanisms of m6A RNA methylation in esophageal cancer: a comprehensive review

**DOI:** 10.3389/fgene.2025.1561799

**Published:** 2025-04-22

**Authors:** Long Wen, Jiang Fu, Zixu Wang, Rangping Xie, Shengjie Tang, Li Yu, Haining Zhou

**Affiliations:** ^1^ Department of Thoracic Surgery, Suining Central Hospital, An Affiliated Hospital of Chongqing Medical University, Suining, China; ^2^ Graduate School, North Sichuan Medical College, Institute of Surgery, Nanchong, China; ^3^ Graduate School, Institute of Surgery, Chengdu University of Traditional Chinese Medicine, Chengdu, China; ^4^ Department of Physical Examination, Suining Central Hospital, An Affiliated Hospital of Chongqing Medical University, Suining, China; ^5^ Key Laboratory of Gastrointestinal Cancer (Fujian Medical University), Ministry of Education, School of Basic Medical Sciences, Fujian Medical University, Fuzhou, China

**Keywords:** esophageal cancer, RNA modification, N6-methyladenosine, occurrence, treatment

## Abstract

Esophageal cancer is an aggressively malignant neoplasm characterized by a high mortality rate. Frequently diagnosed at an advanced stage, it presents challenges for optimal therapeutic intervention due to its non-specific symptoms, resulting in lost opportunities for effective treatment, such as surgery, radiotherapy, chemotherapy and target therapy. The N6-methyladenosine (m6A) modification represents the most critical post-transcriptional modification of eukaryotic messenger RNA (mRNA). The reversible m6A modification is mediated by three regulatory factors: m6A methyltransferases, demethylating enzymes, and m6A recognition proteins. These components identify and bind to specific RNA methylation sites, thereby modulating essential biological functions such as RNA processing, nuclear export, stability, translation and degradation, which significantly influence tumorigenesis, invasion, and metastasis. Given the importance of m6A modification, this paper offers a comprehensive examination of the regulatory mechanisms, biological functions, and future therapeutic implications of m6A RNA methylation in the context of esophageal cancer.

## 1 Introduction

Esophageal cancer ranks among the most prevalent malignant tumors of the digestive tract, positioned 11th in incidence and seventh in mortality worldwide. In 2022, there were 510,716 new cases of esophageal cancer and 445,129 deaths from the disease (Bray et al., 2024). This malignancy predominantly manifests in two pathological forms: esophageal squamous cell carcinoma (ESCC) and esophageal adenocarcinoma (EAC), with ESCC constituting over 90% of all cases (Pennathur et al., 2013; Lin et al., 2014; Blot et al., 1991). Neoadjuvant therapy constitutes a multimodal treatment strategy administered prior to surgical intervention for esophageal cancer. This approach seeks to diminish tumor size and eradicate micrometastases via the combined modalities of chemotherapy, radiotherapy and immunotherapy thereby enhancing both the likelihood of successful surgery and patients’ long-term survival rates. By combining the cytotoxic effects of chemotherapy and the localized impact of radiotherapy, neoadjuvant chemoradiotherapy (nCRT) facilitates tumor downstaging, increases the likelihood of achieving an R0 resection, and reduces locoregional recurrence. The CROSS trial, a landmark randomized controlled study, revealed that nCRT followed by surgery significantly improved median overall survival compared to surgery alone (49.4 vs. 24.0 months; HR 0.657; 95% CI 0.495–0.871; p = 0.003) (Hagen et al., 2012). A prospective, single-center, open-label, randomized phase III clinical trial found that in resectable ESCC, the addition of perioperative immunotherapy to NAC is safe, may improve OS and might change the standard treatment in the future (94.1% vs. 83.0%; HR = 0.48; 95% CI = 0.24 ∼ 0.97; P = 0.037) (Zheng et al., 2024). Moreover, pathologic complete response (pCR) was observed in approximately 29% of patients treated with nCRT, a factor strongly associated with prolonged survival (Shapiro et al., 2015). Although multimodal treatment strategies—including neoadjuvant chemotherapy or chemoradiotherapy following esophagectomy—have enhanced survival outcomes, the persistent challenges of high local recurrence rates and low long-term survival for patients with locally advanced esophageal cancer continue to undermine treatment efficacy (Watanabe et al., 2020; Leng et al., 2021; Guo and Fang, 2021). This predicament arises largely from the absence of overt symptoms in the early stages of the disease and the lack of highly sensitive, specific biological markers, leading most patients to receive a diagnosis only at intermediate or advanced stages. Consequently, there is an urgent need to identify biomarkers with both high specificity and sensitivity to facilitate early diagnosis and improve prognostic assessments.

Transcriptional abnormalities in genes associated with esophageal cancer, encompassing chromosomal and tumor cell mutations, have been a focal point of research in recent years (Chen W. et al., 2021), studies indicate that gene mutations represent a significant mechanism underlying the development of esophageal cancer, with numerous mutations identified in affected patients. Among these, the TP53 mutation is the most prevalent, while other frequently mutated genes include CCND1, CDK4/CDK6, MDM2, CCNE1, cyclin E, and MGST1 (Huang and Yu, 2018; Buas et al., 2017). Beyond epigenetic alterations induced by gene mutations, the emergence of chemoresistance in esophageal cancer has garnered increasing attention. Research into the plasticity of esophageal cancer cells and their resistance to chemotherapy has shown that abnormal epigenetic modifications play a crucial role in phenotypic changes (Zhao and Casson, 2008; Zhang et al., 2021; Kit et al., 2016). Unlike abnormal gene transcription, epigenetic modifications are reversible, making them a promising area of research.

N6-methyladenosine (m6A) modification, one of the most prevalent chemical modifications of mRNA in eukaryotic cells, significantly influences tumor development and progression. Growing evidence implicates m6A in various regulatory mechanisms associated with programmed cell death (Zhao and Casson, 2008),metabolism (Zhang et al., 2021), drug resistance (Zhang et al., 2021), expression of oncogenes and tumor suppressor genes (Zhang et al., 2021), immunotherapy (Zhao et al., 2020) and targeted therapy (Muthusamy, 2020) as part of various regulatory mechanisms. The most common modification of mRNA, m6A methylation, predominantly occurs in the 3′untranslated region near the mRNA terminator and within the long exon regions. Additionally, m6A modifications can be observed in pre-RNA and long non-coding RNAs (lncRNAs). Non-coding RNAs—including microRNAs (miRNAs), lncRNAs, and circular RNAs (circRNAs)—play essential roles in genome transcription and various biological functions at the RNA level. Notably, stable expression of non-coding RNAs can serve as potential biomarkers for cancer diagnosis, prognosis, and clinical treatment. Recent studies have illuminated the influence of m6A modification not only on mRNA regulation but also on the production and functionality of non-coding RNAs such as miRNAs, lncRNAs, and circRNAs. These non-coding RNAs regulate critical cellular biological functions, including proliferation, invasion, and metastasis of specific tumor cells. Furthermore, non-coding RNAs modified by m6A also exhibit regulatory capabilities (Sung et al., 2021; Aguilo et al., 2015; Choe et al., 2018; Wang et al., 2016; Chen X. et al., 2021; Liao et al., 2022; Liang et al., 2021). Thus, the interplay between m6A and non-coding RNAs may offer synergistic opportunities in cancer treatment.

This review summarizes the research results of m6A methylation modification and esophageal cancer, and describes the composition, mode of action, biological function in the progression of esophageal cancer, as well as the treatment, diagnostic value and potential clinical application of m6A methylation modification in esophageal cancer. This study provides a theoretical basis for further exploring the mechanism of the occurrence and development of esophageal cancer, and searching for biomarkers and therapeutic targets for esophageal cancer.

## 2 The mechanism and function of m6A methylation

**FIGURE 1:**
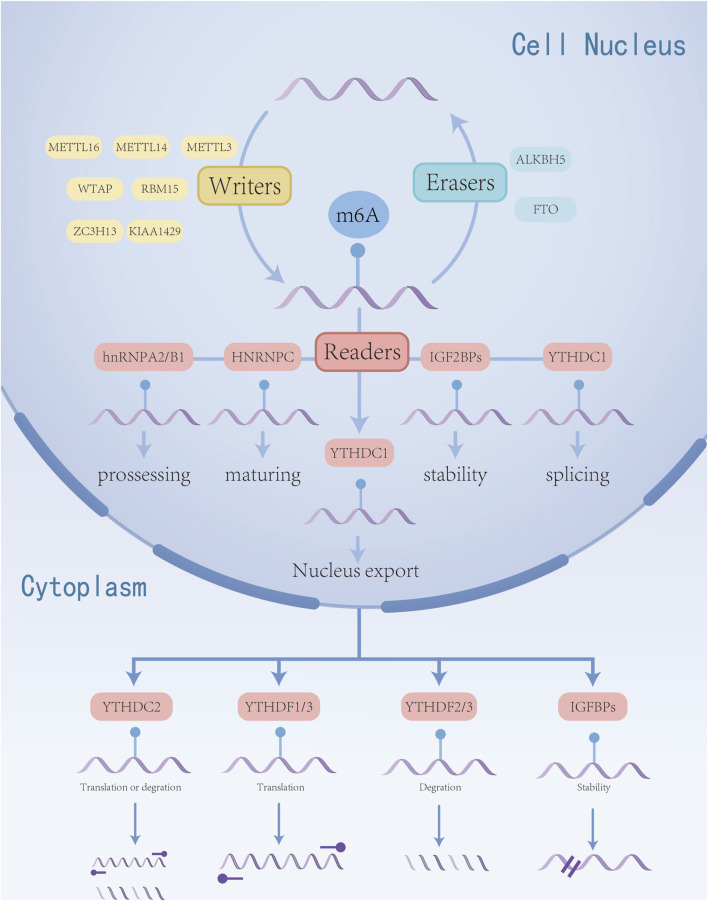
Regulation mechanism of m6A RNA methylation in esophageal cancer. m6A modification is regulated by “writers” and “erasers”. m6A can be recognized by various “reader” proteins, thereby regulating the biological functions of RNA.

The m6A methylation modification is characterized by the transfer of a methyl group from S-adenosyl methionine (SAM) to the N6 position of adenosine, representing a dynamic and reversible modification process, this modification is primarily regulated by the m6A methyltransferase complex (often referred to as “writers”), m6A demethylases (known as “erasers”), and m6A recognition proteins (termed “readers”). It is mainly concentrated in the 3’untranslated region near the mRNA terminator and occurs in the RRm6ACH sequence (Kane and Beemon, 1985). m6A plays a crucial role in various cellular RNA processes, including transcription, splicing, nuclear trafficking, translation, and degradation (Liu et al., 2015; Wang et al., 2014). By influencing the stability and half-life of mRNA, m6A governs gene expression and regulates essential biological functions such as mammalian reproductive processes, circadian rhythms, adipogenesis, and human lifespan (Parashar et al., 2018). m6A methylation adjustment factor has been confirmed that the abnormal expression in a variety of tumors, in cell proliferation, invasion, metastasis and malignant biological line to play an important role in regulating (Zhao et al., 2020) and participate in leukemia, glioblastoma, lung cancer, esophageal cancer and other cancer occurrence and development process (Muthusamy, 2020). The basic mechanism of m6A methylation in RNA is shown in Figure 1.

### 2.1 Methyltransferase

Methyltransferases are also called “writers” and are typically found in the form of methyltransferase complexes. The complexes are mainly composed of methyltransferase-like 3 (METTL3), methyltransferase-like 4 (METTL14), auxiliary factor Wilms’ tumor 1-related protein (WTAP), viral-like m6A methyltransferase-related protein (VIRMA/KIAA1429), zinc finger CCCH domain protein 13 (ZC3H13) and so on (Lan et al., 2019; Wang X. et al., 2021; Gong et al., 2020). Among these, METTL3, METTL14, and WTAP serve as the core catalytic components. METTL3 acts as a pivotal catalytic enzyme, capable of binding S-adenosylmethionine and transferring a methyl group to the N6 position of adenosine. Additionally, cytoplasmic METTL3 can target the 3′non-coding region of m6A-modified mRNAs, facilitating the recruitment of eukaryotic translation initiation factor 3 subunit H (EIF3H) to enhance translation efficiency. Notably, the expression levels of METTL3 correlate with various clinical parameters in esophageal cancer, including tumor size, invasion depth, degree of differentiation, lymph node metastasis, distant metastasis, and TNM staging (Chen X. et al., 2021; Liao et al., 2022; Liang et al., 2021). Similarly, WTAP expression is linked to TNM staging and lymph node metastasis Zou et al. (2024) demonstrated that protein tyrosine phosphatase type IVA member 1 (PTP4A1) is an m6A target of WTAP. Specifically, WTAP positively regulates the expression of PTP4A1 and activates the AKT-mTOR signaling pathway, thereby promoting the proliferation of ESCC cells through the regulation of PTP4A1 expression. Further investigations have identified additional methyltransferases, such as methyltransferase-like proteins 5 and 16 (METTL5 and METTL16), which mediate m6A modification at specific RNA target sites (Pendleton et al., 2017; van Tran et al., 2019). Rong et al. (2020) demonstrated that METTL5 can catalyze m6A modification in the A1832 region of 18S rRNA, as confirmed through high-performance liquid chromatography-mass spectrometry (HPLC-MS) analysis. Additionally, researchers including (Brown et al., 2016; Warda et al., 2017) Jessica et al. have identified METTL16 via mass spectrometry, suggesting the existence of a hypothetical RNA methyltransferase that regulates U6 snRNA, various non-coding RNAs, numerous long non-coding RNAs (lncRNAs), and pre-mRNA m6A modification. Recently, zinc finger CCHC domain protein 4 (ZCCHC4) has been discovered as a novel methyltransferase primarily responsible for methylating human 28S rRNA and some certain mRNAs (Ma et al., 2019).

### 2.2 Demethylases

Demethylases are also called “erasers,” and this enzyme can remove the m6A modification from RNA molecules, which is a key point in the reversible m6A modification process (Ma et al., 2019). To date, we have only discovered two demethylases, one is the obesity-related gene (FTO), and the other is alpha-ketoglutarate-dependent dioxygenase homolog 5 (ALKBH5) (Zhao et al., 2014; Zheng et al., 2013). Both of them can remove m6A modification from RNA or DNA. Iron and alpha-ketoglutarate are auxiliary factors for FTO and ALKBH5, which catalyze the formation of their biological functions. ALKBH5 plays a dual role in various cancers by regulating proliferation, migration, invasion, metastasis, and tumor growth through various biological processes. For example, Dong et al. indicated a positive correlation between ALKBH5 expression and advanced esophageal cancer; conversely, its overexpression significantly diminished the migration and invasion capabilities of esophageal cancer cells (Xiao et al., 2021). Li et al. demonstrated that increased ALKBH5 expression led to reduced overall cellular m6A methylation levels, subsequently inhibiting malignant proliferation and invasion (Li J. et al., 2021). FTO, on the other hand, interacts with HSD17B11 mRNA, decreasing its m6A methylation levels and, through YTHDF1, influencing its translation. Elevated HSD17B11 levels have been associated with enhanced lipid droplet formation and progression of esophageal cancer (Duan et al., 2022). FTO also upregulates both mRNA and protein levels of MMP13, promoting the proliferation and migration of esophageal cancer cells (Liu S. et al., 2020). Moreover, FTO promotes tumor growth in esophageal cancer by demethylating m6A sites on long non-coding RNA LINC00022. This lncRNA directly binds to the p21 protein, facilitating its ubiquitination-mediated degradation, thereby accelerating cell cycle progression and proliferation. Notably, increased expression of FTO in esophageal cancer results in diminished m6A methylation of the LINC00022 transcript, inhibiting its degradation via the m6A reader YTHDF2. Consequently, FTO overexpression can enhance LINC00022-dependent cell proliferation and tumor growth in esophageal cancer (Cui et al., 2021).

### 2.3 Binding proteins

m6A binding proteins are also called “readers”, m6A modifications are recognized by readers, facilitating their binding and subsequent participation in downstream processes, including translation, mRNA degradation, and nucleation acceleration including YT521-B family of homologous proteins (YTHDC1, YTHDC2, YTHDF1, YTHDF2, and YTHDF3), insulin-like growth factor 2 mRNA binding protein (IGF2BP1, IGF2BP2, IGF2BP3), heterogeneous ribonucleoprotein (HNRNP) and eukaryotic translation initiation factor 3 (EIF3) (Xu et al., 2014; Zhu et al., 2014; Zheng et al., 2021). However, different “readers” can produce different biological effects. In the YTH domain family, YTHDF1 binds initiation factors to enhance mRNA translation and protein synthesis (Wang et al., 2015). Zhang L. et al. (2024) discovered that YTHDF1 expression was significantly upregulated in ESCA and associated with poor prognosis. They also demonstrated that TINAGL1 might be a potential target of YTHDF1. YTHDF1 recognized and bound to m6A-modified sites on TINAGL1 mRNA, resulting in enhanced translation of TINAGL1. Furthermore, TINAGL1 knockdown partially mitigated the tumor-promoting effects of YTHDF1 overexpression. Thus, they revealed that YTHDF1 facilitates ESCA progression by promoting TINAGL1 translation in an m6A-dependent manner, offering an attractive therapeutic target for ESCA. In contrast, YTHDF2 facilitates transcript degradation by selectively binding to m6A-modified mRNA and directing it to decay sites (Wang et al., 2014). Recent studies have found that B-cell lymphoma-2-associated transcription factor 1 (BCLAF1) is overexpressed in ESCC tissues. YTHDF2, as a key protein interacting with BCLAF1, it’s tumor suppressor activity of YTHDF2 can be reduced by BCLAF1 (Zhang P. et al., 2024). YTHDF3 contributes to the regulation of RNA translation through its interaction with YTHDF1, while also promoting RNA degradation by associating with YTHDF2 (Duan et al., 2022). Additionally, insulin-like growth factor binding proteins (IGFBPs) are known to enhance RNA expression by stabilizing RNA molecules, thereby increasing their stability and availability for translation (Li et al., 2019). The roles and classification of m6A enzymes in RNA metabolism are detailed in Table 1.

**TABLE 1:** Shows the functions of the various enzymes.

Type	Enzyme	Function	Reference
Writers	Mettl3/Mettl14	Form a stable heterodimer core complex that functions in cellular m6A deposition on mammalin nuclear RNAs	Liu et al. (2014)
	Mettl16	Catalyze m6A modification of U6 snRNA and other lncRNAs	Satterwhite and Mansfield (2022)
	WTAP	A regulatory subnuit, recruits the m6A methyltransferase complex to the target mRNA and ensures the heterodimer located on the nuclear spot and promotes the catalytic activity	Huang et al. (2022)
	RBM15/RBM15B	Binds the m6A complex and recruit it to special RNA site	Jiang et al. (2021)
	ZC3H13	Promote methyltransferase complex RNA binding	Knuckles et al. (2018)
Erasers	FTO	The first m6A demethylase	Li et al. (2022)
	ALKBH5	Remove m6A modification from RNA or DNA	Zheng et al. (2013)
Readers	YTHDF1	Enhance mRNA translation and protein synthesis	Wang et al. (2015)
	YTHDF2	Induce transcript degradation	Wang et al. (2014)
	YTHDF3	Enhance RNA translation by interacting with YTHDF1 and promote RNA degradation by binding to YTHDF2	Shi et al. (2017)
	YTHDC1	Function in alternative splicing and mRNA export	Xiao et al. (2016)
	YTHDC2	A probable ATP-dependent RNA helicase that promotes mRNA translation	Wojtas et al. (2017)
	IGF2BPs	Protect mRNA transcripts from degradation and modulate the alternative splicing of mRNA	Huang et al. (2018)
	hnRNP A2/B1	It binds and controls the processing of nascent RNA and is also involved in telomere maintenance and DNA repair	Liu and Shi (2021)
	eIF3H	Initiate protein translation, regulate the selective translation of mRNAs and facilitate ribosome assembly	Choe et al. (2018)

## 3 The role and significance of m6A methylation in EC

Studies have demonstrated that the expression of m6A-modified mRNA is dysregulated across various cancers, with both *in vivo* and *in vitro* experiments confirming the role of m6A in tumor biology, For example, FTO rearrangement in t (11q23)/MLL, t (15; 17)/PML-RARA, FLT3-ITD and NPM1 mutated AML, which promoted leukemic cell transformation and tumorigenesis (Li Z. et al., 2017), Additionally, METTL3 and METTL14 can inhibit the growth and self-renewal of glioblastoma stem-like cells (GSCs) (Zhang et al., 2017), In lung cancer, METTL3 functions as an oncogene by enhancing the expression of EGFR and TAZ, thereby promoting cell growth, survival, and invasion (Lin S. et al., 2016), furthermore, METTL3 has been linked to poor prognosis in hepatocellular carcinoma (HCC) patients, facilitating HCC cell proliferation, migration, and colony formation through YTHDF2-dependent post-transcriptional silencing of SOCS2 (Chen et al., 2018). Both overexpression and depletion of these m6A-related factors can significantly alter m6A modification in tumors, influencing cancer progression. Elucidating the molecular mechanisms of m6A-modified RNA and identifying abnormal expression in clinical biopsy specimens is of paramount importance for the early diagnosis of tumors, predicting tumor prognosis, and developing novel therapeutic strategies.

### 3.1 The effect of m6A modification on mRNA in EC

**FIGURE 2:**
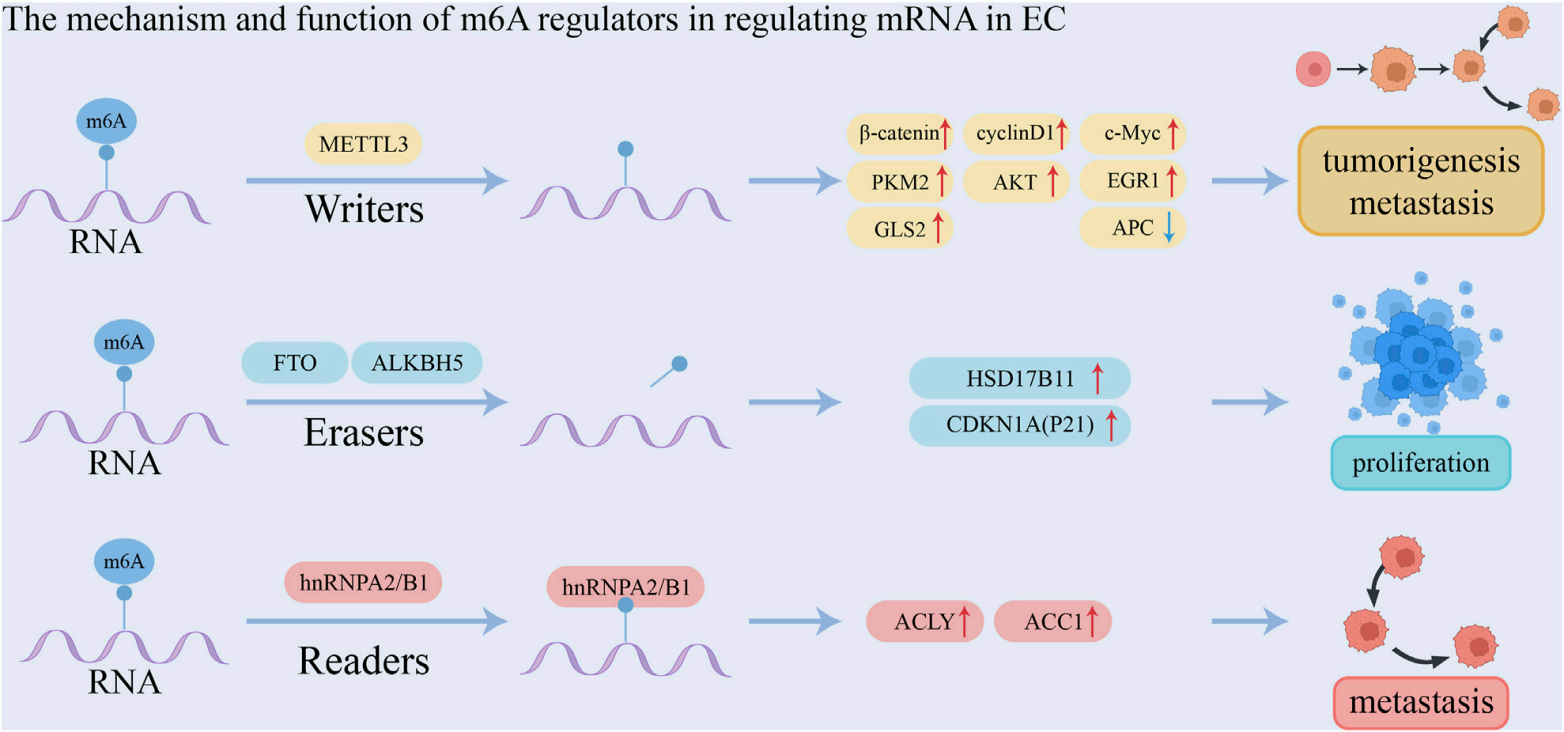
The mechanism and function of m6A regulators in regulating mRNA in EC.

The effect of m6A modification on mRNA in ESCA is mediated by m6A readers that bind to it. The YTH family of proteins includes YTH m6A-binding protein 1 (YTHDF1), YTHDF2, YTHDF3, YTH domain-containing protein 1 (YTHDC1), and YTHDC2, all of which possess a specific YTH domain that enables them to recognize and bind to target RNAs in an m6A-dependent manner (Xu et al., 2014; Zhu et al., 2014). YTHDF1 promotes translation initiation and protein synthesis (Wang et al., 2015), whereas YTHDF2 facilitates m6A-modified mRNA degradation by either directing m6A-modified mRNAs to degradation sites or recruiting the CCR4-NOT deadenylase complex to initiate degradation (Wang et al., 2014; Du et al., 2016). YTHDF3 interacts with YTHDF1 or YTHDF2, exerting dual effects by either promoting mRNA translation or enhancing degradation (Shi et al., 2017; Li A. et al., 2017). YTHDC1 plays a role in exon splicing and promotes the nuclear export of m6A-modified mRNAs to the cytoplasm (Xiao et al., 2016; Roundtree et al., 2017; Lesbirel et al., 2018). YTHDC2, on the other hand, enhances the translation efficiency of m6A-modified mRNAs, thereby reducing their abundance (Wojtas et al., 2017; Hsu et al., 2017). IGF2BPs, including IGF2BP1, IGF2BP2, and IGF2BP3, are known to increase mRNA stability (Huang et al., 2018). Studies revealed that RPS15 interacts with the K homology domain of IGF2BP1, which recognizes and binds the 3′-UTR of MKK6 and MAPK14 mRNA in an m6A-dependent manner, promoting translation of p38 MAPK pathway proteins. Through virtual screening and functional assays, folic acid was found to target RPS15, exhibiting therapeutic effects on ESCC, enhanced when combined with cisplatin. Inhibition of RPS15 (e.g., by folic acid, IGF2BP1 ablation, or SB203580 treatment) suppressed ESCC metastasis and proliferation via the p38 MAPK pathway. Thus, RPS15 drives ESCC progression through this pathway, and RPS15 inhibitors may serve as potential anti-ESCC therapeutics (Zhao et al., 2023). Heterogeneous nuclear ribonucleoproteins (HNRNPs), such as HNRNPA2B1, HNRNPC, and HNRNPG, regulate alternative splicing of mRNAs in an m6A-dependent manner (Liu et al., 2015; Liu et al., 2017). EIF3 functions as an m6A reader within the 5′-UTR of mRNA and plays a pivotal role in nearly all stages of translation initiation, a key rate-limiting step in protein synthesis. It facilitates the assembly of the 43S preinitiation complex (PIC), mediates the interaction between the 43S PIC and mRNA via the EIF4F complex, and aids in scanning for the AUG initiation codon (Pestova et al., 2001; Jackson et al., 2010; Sokabe and Fraser, 2014; Pestova and Kolupaeva, 2002). In addition, Wang W. et al. (2021) conducted a study involving 200 patients with esophageal squamous cell carcinoma (ESCC) and reported that METTL3 was upregulated in tumor tissues. METTL3 recruited YTHDF to facilitate the degradation of adenomatous polyposis coli (APC) mRNA by enhancing the m6A modification of the APC gene. This led to a reduction in APC expression and an increase in the expression of β-catenin, cyclin D1, C-Myc, and PKM2, thereby promoting aerobic glycolysis, proliferation, and metastasis of ESCC cells. Furthermore, METTL3 was found to promote ESCC metastasis by upregulating glutaminase-2 (GLS2) expression. This study was the first to demonstrate that GLS2 is regulated by METTL3 through m6A modification. Thus, the METTL3/GLS2 signaling pathway may serve as a potential therapeutic target for anti-metastasis strategies in ESCC. Hou et al. (2020) found that METTL3-mediated activation of the AKT signaling pathway promotes the initiation and progression of esophageal cancer. Upregulation of METTL3 enhances the proliferation, migration, and invasion of ESCC cells while inhibiting apoptosis. Additionally, METTL3 increases the m6A modification of EGR1 mRNA in a YTHDF3-dependent manner, enhancing its stability and activating the EGR1/Snail signaling pathway (Liao et al., 2022). Therefore, METTL3 is upregulated in esophageal cancer, promotes proliferation and metastasis, and may serve as an independent prognostic biomarker (Xia et al., 2020). m6A influences mRNA splicing by recruiting hnRNPA2B1 or altering local structure, increasing the accessibility of splicing factors like hnRNPC, which affects transcription (Zhou et al., 2019). Guo et al. (2020) using The Cancer Genome Atlas data, found that m6A levels, along with regulatory factors ALKBH5 and hnRNP A2/B1, are significantly upregulated in esophageal cancer patients, indicating a potential role in prognosis. Upregulation of hnRNP A2/B1 promotes esophageal cancer proliferation and metastasis by enhancing the expression of oncogenic factors ACLY and ACC1. Therefore, ALKBH5 and hnRNPA2B1 may serve as diagnostic and prognostic markers, as well as therapeutic targets. FTO also promotes lipid droplet formation in esophageal cancer cells via upregulation of HSD17B11, contributing to tumor proliferation and invasion (Duan et al., 2022). Nagaki et al. (2020) reported that upregulation of ALKBH5 is associated with poor prognosis in esophageal cancer. Inhibition of ALKBH5 delayed the esophageal cancer cell cycle, causing cell arrest at the G0/G1 phase. In ALKBH5-deficient cells, CDKN1A (P21) expression was significantly increased, and ALKBH5 knockdown led to enhanced m6A modification and greater CDKN1A mRNA stability. The mechanism and function of m6A regulators in regulating mRNA in EC is shown in Table 2 and Figure 2.

**TABLE 2:** Shows various m6A regulators in regulating mRNAs in esophageal cancer.

m6A regulators	Target	Location	Role	Mechanism	Function	Reference
Mettl3	APC	mRNA	Oncogene	Degrade APC mRNA	Promoting EC tumorigenesis and metastasis	Wang et al. (2021b)
	β-catenin	mRNA	Oncogene	Enhance expression of β-catenin	Promoting EC tumorigenesis and metastasis	Wang et al. (2021b)
	cyclin D1	mRNA	Oncogene	Enhance expression of cyclin D1	Promoting EC tumorigenesis and metastasis	Wang et al. (2021b)
	c-Myc	mRNA	Oncogene	Enhance expression of c-Myc	Promoting EC tumorigenesis and metastasis	Wang et al. (2021b)
	PKM2	mRNA	Oncogene	Enhance expression of PKM2	Promoting EC tumorigenesis and metastasis	Wang et al. (2021b)
	AKT	mRNA	Oncogene	Enhance expression of AKT	Promoting EC tumorigenesis and metastasis	Wang et al. (2021b)
	EGR1	mRNA	Oncogene	Enhance the stability of EGR1-mRNA	Promoting EC tumorigenesis and metastasis	Wang et al. (2021b)
	GLS2	mRNA	Oncogene	Enhance expression of GLS2	Promoting EC tumorigenesis and metastasis	Wang et al. (2021b)
hnRNP A2/B1	ACLY and ACC1	mRNA	Oncogene	Enhance expression of ACLY and ACC1	Promoting EC metastasis	Guo et al. (2020)
FTO	HSD17B11	mRNA	Oncogene	Upregulate HSD17B11	Promoting EC proliferation	Duan et al. (2022)
ALKBH5	CDKN1A (p21)	mRNA	Oncogene	Degrade CDKN1A (p21) mRNA	Promoting EC proliferation	He et al. (2023)

### 3.2 The effect of m6A modification on non-coding RNAs in EC

**FIGURE 3:**
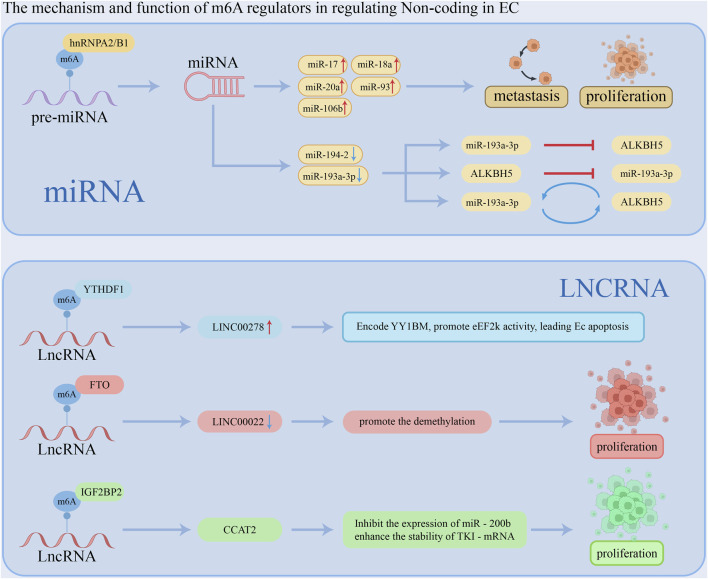
The mechanism and function of m6A regulators in regulating Non-coding RNAs in EC.

Non-coding RNAs (ncRNAs), including miRNA, lncRNA, and circRNA, lack coding potential; however, they play a crucial role in the regulation of gene expression. The mechanism and function of m6A regulators in regulating Non-coding RNAs in EC is shown in Figure 3.

#### 3.2.1 microRNA

MicroRNAs (miRNAs) are a class of endogenous small RNAs, typically measuring 20 to 24 nucleotides in length, that play crucial regulatory roles within cells by participating in gene silencing and post-transcriptional regulation of gene expression (Kong et al., 2020). miRNAs bind to the 3′untranslated region (3′UTR) of target mRNAs, leading to the silencing or repression of the corresponding genes. The biogenesis of miRNAs involves several key steps. Initially, primary miRNAs (pri-miRNAs) are transcribed from DNA. These pri-miRNAs are then processed into precursor miRNAs (pre-miRNAs) by a microprocessor complex that includes Drosha ribonuclease III (Drosha) and DiGeorge syndrome critical region 8 (DGCR8). Finally, pre-miRNAs are further cleaved to produce mature miRNAs. In ESCC, the m6A writer and m6A eraser deposit and delete m6A on pri-miRNA, respectively (Liang et al., 2021; Liu et al., 2021a; Chen P. et al., 2021; Li K. et al., 2021). For example, Li et al. analyzed esophageal cancer data from the TCGA database and found that the expression levels of 25 m6A regulators were elevated and positively correlated. Notably, increased expression of hnRNPA2B1 was associated with lymph node metastasis in esophageal cancer and correlated with poor prognosis. Additionally, knockdown of hnRNPA2B1 significantly reduced the expression of miR-17, miR-18a, miR-20a, miR-93, and miR-106b, leading to decreased proliferation and metastasis of esophageal cancer cells (Li K. et al., 2021). Moreover, METTL3 was shown to elevate the m6A level of pri-miR-200-5p, while ALKBH5 reduced the m6A level of pri-miR-194-2 (Liang et al., 2021; Chen P. et al., 2021). Xue et al. (2021) demonstrated that miR-193a-3p was upregulated in ESCC tumor tissues compared to normal tissues, promoting invasion, metastasis, and recurrence. Additionally, miR-193a-3p and ALKBH5 regulate each other in a feedback loop, affecting the progression of ESCC. The mechanism and function of m6A regulators in regulating MicroRNA in ESCC is shown in Table 3.

**TABLE 3:** Shows various m6A regulators in regulating miRNAs in esophageal cancer.

m6A regulators	Target	Location	Role	Mechanism	Function	Reference
hnRNP A2/B1	miR-17	miRNA	Oncogene	Enhance expression of miR-17	Promoting EC proliferation and metastasis	Li et al. (2021b)
	miR-18a	miRNA	Oncogene	Enhance expression of miR-18a	Promoting EC proliferation and metastasis	Li et al. (2021b)
	miR-20a	miRNA	Oncogene	Enhance expression of miR-20a	Promoting EC proliferation and metastasis	Li et al. (2021b)
	miR-93	miRNA	Oncogene	Enhance expression of miR-93	Promoting EC proliferation and metastasis	Li et al. (2021b)
	miR-106b	miRNA	Oncogene	Enhance expression of miR-106b	Promoting EC proliferation and metastasis	Li et al. (2021b)
Mettl3	miR-200-5p	miRNA	Oncogene	Enhance expression of miR-200-5p	Inhibit Nuclear Factor I-C transcription and promote EMT, invasion and migration	Liang et al. (2021)
ALKBH5	miR-194-2	miRNA	Tumor suppressor	Decrease m6A level of pri-miR-194-2	Inhibit the proliferation of ESCC	Chen et al. (2021c)
ALKBH5	miR-193a-3p	miRNA	Oncogene	miR-193-3p target alKBH5 and suppress its expression, ALKBH5 inhibit miR-193a-3p expression in turn	Promoting EC proliferation and metastasis	Xue et al. (2021)

#### 3.2.2 lncRNA

Long non-coding RNAs (lncRNAs) are a class of endogenous RNA molecules defined as non-coding RNAs longer than 200 nucleotides. They have been reported to play a significant role in various diseases, including esophageal squamous cell carcinoma. m6A modification is also present on lncRNAs, potentially influencing gene expression by affecting the interaction of lncRNAs with RNA-binding proteins through an “m6A switch” mechanism or by modulating the interaction between lncRNAs and miRNAs (Liu et al., 2015; Yang et al., 2018). Wu et al. (2020) identified a Y-linked lncRNA, LINC00278, which is downregulated in male EC. LINC00278 encodes a micropeptide, YY1BM, that binds to the Yin Yang 1 (YY1) protein and disrupts its interaction with the androgen receptor (AR), leading to reduced expression of eEF2K through the AR signaling pathway. When YY1BM is downregulated, eEF2K levels rise, reducing apoptosis and making ESCC cells more resistant to nutrient deprivation. Cigarette smoking inhibits LINC00278's m6A modification, decreasing YY1BM production and impacting AR signaling. Moreover, smoking decreased the m6A-modified LINC00278 and YY1BM translation. Another study (Cui et al., 2021) demonstrated that lncRNA LINC00022 is upregulated in esophageal squamous cell carcinoma (ESCC). FTO demethylates LINC00022, inhibiting its degradation and thereby promoting tumor growth. In ESCC cells and tissues, IGF2BP2, TK1, and lncRNA CCAT2 are also upregulated, while miR-200b expression is reduced. CCAT2 binds to miR-200b, decreasing its levels and leading to increased expression of IGF2BP2. IGF2BP2 stabilizes TK1 mRNA by recognizing its m6A modification, which enhances TK1 expression and facilitates the migration and invasion of ESCC cells (Wu et al., 2021). The mechanism and function of m6A regulators in regulating lncRNA in ESCC is shown in Table 4.

**TABLE 4:** Shows various m6A regulators in regulating lncRNAs in esophageal cancer.

m6A regulators	Target	Location	Role	Mechanism	Function	Reference
YTHDF1	LINC00278	lncRNA	Tumor suppressor	Enhance expression of LINC00278	Encoding YYIBM, promoting eEF2 activity, leading to EC apoptosis	Wu et al. (2020)
FTO	LINC00022	lncRNA	Oncogene	Promote the demethylation of LINC00022	Promote the proliferation of EC	Cui et al. (2021)
IGF2BP2	CCAT2	lncRNA	Oncogene	Inhibit the expression of miR-200b, enhance the stability of TK1-mRNA	Promote the proliferation of EC	Wu et al. (2021)

#### 3.2.3 circRNA

Circular RNAs (circRNAs) represent a novel class of non-coding RNAs that form covalently closed continuous loops through a process known as reverse splicing, distinguishing them from linear RNAs. These circRNAs perform multiple biological functions, largely dependent on their specific cellular distribution. Nuclear circRNAs can influence transcription and splicing (Yang L. et al., 2020; Conn et al., 2017). In the cytoplasm, circRNAs can act as sponges to absorb microRNAs, reducing their ability to suppress target mRNAs, thereby enhancing gene expression (Thomson and Dinger, 2016; Hansen et al., 2013). Recent studies have revealed that m6A modification is prevalent in circRNAs, exhibiting a read-write mechanism akin to that observed in mRNAs (Zhou et al., 2017). Wang et al. (2020) analyzed plasma samples from 10 EC patients across different TNM stages, as well as samples from 5 healthy controls, to investigate circRNA expression profiles. They found that levels of plasma circ-SLC7A5 correlated with TNM staging. Circ-SLC7A5 contained numerous m6A modification sites, indicating a high potential for translation. Furthermore, it demonstrated a strong affinity for binding to open reading frames and harbored a higher number of microRNA-recognition elements (MREs), indicating that circRNAs may play multifaceted roles in EC. Unfortunately, to date, no other studies have investigated how m6A modification regulates the expression and function of circRNAs in the development and progression of esophageal cancer, highlighting an important avenue for future research.

## 4 Expression of m6A modification related proteins in ESCA

### 4.1 ALKBH5

Previous studies have indicated that ALKBH5 expression is diminished in esophageal cancer (EC) tissues, with functional analyses demonstrating that ALKBH5 inhibits the proliferation, migration, and invasion of EC cell. However, a recent investigation suggested that ALKBH5 may instead promote the proliferation and migration of EC (Yang N. et al., 2020). This finding contradicts earlier results, potentially due to the absence of a comparative analysis between ESCC and normal esophageal tissues; the recent study focused solely on ALKBH5 expression within ESCC. Data from The Cancer Genome Atlas (TCGA), analyzed using the online tool GEPIA, reveal that patients with high ALKBH5 expression exhibit longer overall survival compared to those with low expression, suggesting a tumor-suppressive role for ALKBH5 in EC. This protein is implicated in regulating various processes, including cell proliferation, migration, invasion, tumor progression, metastasis, tumorigenesis, and chemoresistance through its influence on m6A methylation. Xiao et al. (2021) reported reduced expression of ALKBH5, an m6A demethylase, in EC tissue specimens, with more pronounced reductions observed in advanced stages (T3-T4, N1-N3, clinical stages III-IV) and higher histological grades (grade III). This suggests a role for ALKBH5 in the progression of ESCC. Exogenous expression of ALKBH5 was found to inhibit the *in vitro* proliferation of ESCC cells, whereas depletion of endogenous ALKBH5 significantly enhanced proliferation. These results imply that ALKBH5 exerts anti-proliferative effects on EC growth. Furthermore, overexpression of ALKBH5 suppressed tumor growth of Eca-109 cells in nude mice, while depletion of endogenous ALKBH5 accelerated tumor growth in TE-13 cells *in vivo*. The growth-inhibitory effects associated with ALKBH5 overexpression appear to be partially mediated by a G1-phase arrest. Additionally, ALKBH5 overexpression was linked to reduced migration and invasion of ESCC cells *in vitro*.

### 4.2 YTHDC2

Yang et al. (2020b) observed that the expression of YTHDC2 is downregulated in esophageal cancer, based on analyses of relevant databases. In proliferation experiments, low levels of YTHDC2 significantly enhanced cell growth, indicating its potential role as a tumor suppressor in EC tissues. Further enrichment analysis using the Kyoto Encyclopedia of Genes and Genomes (KEGG) pathways revealed that several key pathological pathways associated with ESCC—including the p53 signaling pathway, NF-kappaB signaling pathway, and JAK-STAT signaling pathway—were notably enriched with downregulated genes. This enrichment suggests that the diminished expression of YTHDC2 may promote the proliferation of ESCC cells by disrupting these critical signaling pathways.

### 4.3 METTL3

METTL3 serves as the core catalytic component of the methyltransferase complex. Wang W. et al. (2021) analyzed data from The Cancer Genome Atlas (TCGA), revealing that METTL3 is highly expressed in 200 paired esophageal squamous cell carcinoma (ESCC) specimens compared to adjacent normal tissues. Xia et al. (2020) demonstrated that METTL3 expression is elevated in tumor tissues relative to normal counterparts, and higher levels of METTL3 correlate with poorer survival outcomes. In addition, METTL3 levels serve as an independent predictor of disease-free survival and overall survival in EC patients. Hou et al. (2020) further elucidated that METTL3 promotes the progression of human esophageal cancer through AKT signaling pathways, suggesting its potential as a therapeutic target. In conclusion, METTL3 is a promising biomarker for predicting prognosis in EC.

### 4.4 FTO

A previous study (Duan et al., 2022) utilizing immunohistochemistry and data analysis of cancer tissues and paracancerous tissues from 106 patients with esophageal cancer revealed that FTO expression was upregulated in esophageal cancer cells, serving as a negative prognostic indicator for patients. HSD17B11, a member of the short chain dehydrogenase family, is likely to be a target gene regulated by FTO. FTO enhances lipid droplet (LD) formation in esophageal cancer cells by promoting HSD17B11 expression, thereby facilitating the progression of esophageal cancer. Furthermore, Liu S. et al. (2020) conducted immunohistochemical studies on 80 pairs of esophageal cancer tissues and observed significantly elevated FTO expression compared to normal tissues; upregulation of matrix metallopeptidase 13 (MMP13) was found to promote cell proliferation and metastasis. Long non-coding RNA (lncRNA) plays a crucial role in regulating cell proliferation. Cui et al. (2021) identified LNC00022 as a specific m6A target; LINC00022 directly binds to p21 protein and promotes its ubiquitin-mediated degradation, thus driving cell cycle progression and proliferation. Elevated FTO levels in ESCC cells reduced m6A methylation of LINC00022 transcript, inhibiting its degradation through YTHDF2; ultimately leading to increased LNC00022-dependent cell proliferation and ESCC tumor growth. Therefore, targeting FTO as a demethylase presents potential for novel therapeutic strategies against esophageal cancer.

### 4.5 HNRNPA2B1

Guo et al. (2020) found HNRNPA2B1 levels are significantly increased in EC tissues, with high expression positively correlated to tumor diameter and lymph node metastasis. Functional studies indicated that knockout of the HNRNPA2B1 gene inhibits the proliferation, migration, and invasion of EC cells. From a mechanistic perspective, HNRNPA2B1 facilitates the progression of EC by upregulating the expression of key enzymes involved in fatty acid synthesis, namely ATP citrate lyase (ACLY) and acetyl-CoA carboxylase (ACC1). This indicates that HNRNPA2B1 functions as a carcinogenic factor by enhancing fatty acid synthesis, thereby highlighting its potential as a prognostic biomarker and therapeutic target in esophageal cancer. Moreover, both ALKBH5 and HNRNPA2B1 serve as effective indicators for predicting overall survival (OS) in EC patients. High levels of HNRNPA2B1 and low levels of ALKBH5 are identified as risk factors for survival in ESCC. Notably, the combined assessment of these two factors demonstrates superior predictive capability compared to the use of either factor in isolation.

### 4.6 HNRNPC

Studies have demonstrated that HNRNPC is overexpressed in esophageal squamous cell carcinoma (ESCC) tissues, with its expression inversely correlated with the overall survival of patients. Zhou et al. (2023) identified a novel circular RNA, circ-FIRRE, which acts as a platform to interact with the HNRNPC protein. This interaction stabilizes GLI2 mRNA by directly binding to its 3′-untranslated region (UTR) in the cytoplasm, leading to increased GLI2 protein levels. Consequently, this elevation drives the transcription of target genes such as MYC, CCNE1, and CCNE2, thereby contributing to the progression of EC.

## 5 The diagnostic and therapeutic potential of m6A for the EC

Early studies have demonstrated that METTL3 is highly expressed in tumor tissue, that the higher the expression level of METTL3 is, the shorter the survival time of patients is, and that upregulation of METTL3 in tumor tissue is inversely associated with DFS and OS of patients. Liu XS. et al. (2020) conducted immunohistochemical staining on 57 tumor samples from esophageal cancer (ESCA) patients who underwent PET/CT scans prior to surgery. They evaluated the expression of METTL3, glucose transporter 1 (GLUT1), and hexokinase 2 (HK2) in both tumor and peritumoral tissues, analyzing the relationship between maximum standardized uptake value (SUVmax) and the expression levels of METTL3, HK2, and GLUT1. Their findings indicated that increased 18F-FDG uptake was associated with high METTL3 expression. The mechanism underlying this enhanced 18F-FDG uptake may involve the regulation of GLUT1 and HK2 by METTL3. This suggests that PET/CT may serve as a noninvasive method to monitor METTL3 levels, potentially allowing for the use of PET/CT in conjunction with METTL3 as predictors for esophageal cancer. Li L. et al. (2021) developed a 4-miRNA survival prediction model by integrating data from The Cancer Genome Atlas (TCGA) and Gene Expression Omnibus (GEO) datasets. With the advancement of m6A research, modifications may emerge as promising early detection biomarkers for esophageal cancer.

**FIGURE 4:**
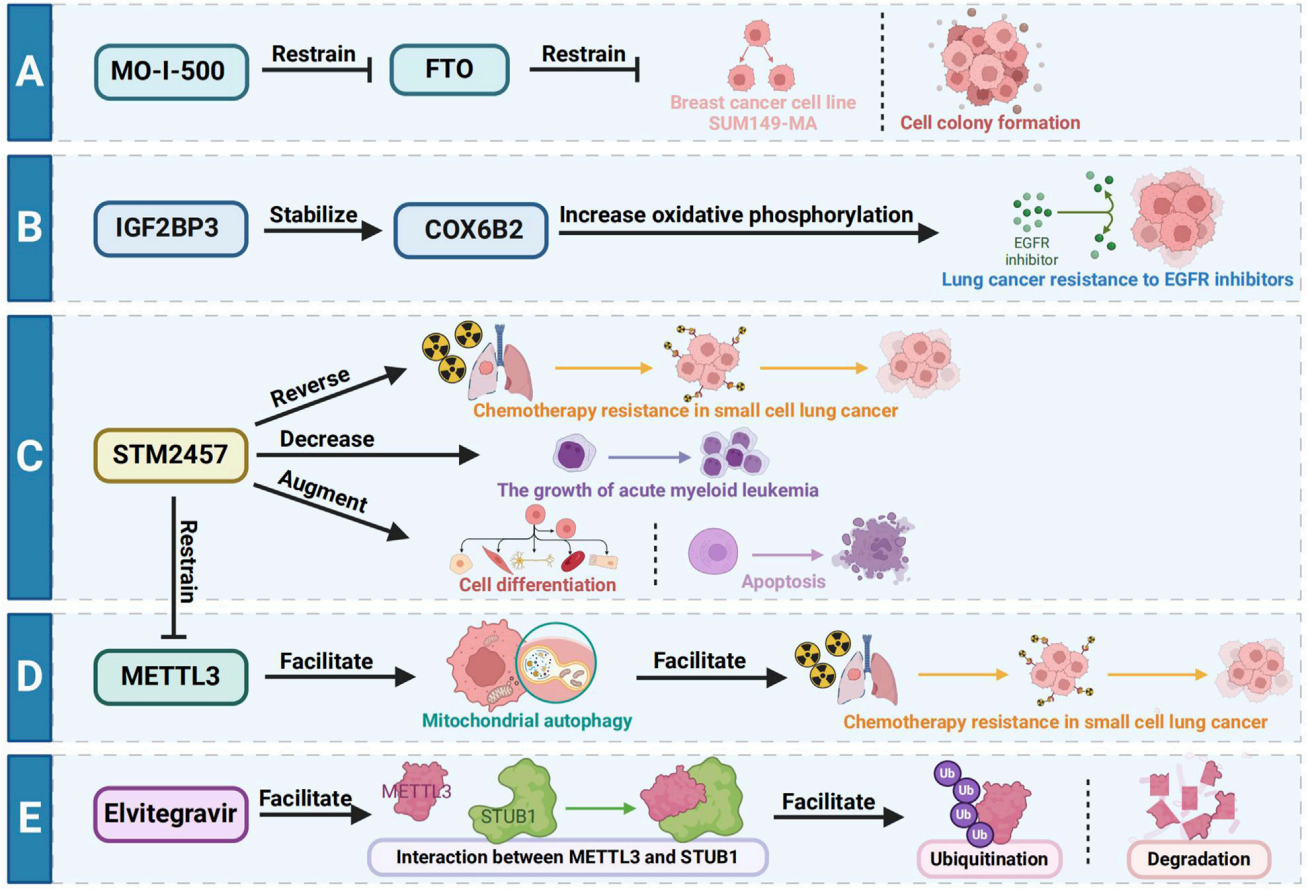
Different therapeutic targets of m6A. **(A)** MO-I-500, as a kind of particular FTO inhibitor, can inhibit the aggressive inflammatory breast cancer cell line SUM149-MA or cell colony formation of survival. **(B)** IGF2BP3 stabilizes COX6B2 to increase oxidative phosphorylation and drive resistance to EGFR inhibitors in lung cancer. **(C)** METTL3 inhibitor STM2457 decreases the growth of acute myeloid leukemia AML and increased differentiation and apoptosis. **(D)** METTL3 promotes chemotherapy resistance in small cell lung cancer by positively regulating mitophagy, while STM2457 could reverse chemotherapy resistance in small cell lung cancer. **(E)** The anti-HIV drug Elvitegravir has been shown to enhance the ubiquitination and subsequent degradation of METTL3 by facilitating its interaction with the E3 ubiquitin ligase STUB1.

The main treatment methods of esophageal cancer include surgery, radiotherapy, chemotherapy and immunotherapy. However, because most patients are diagnosed with esophageal cancer at an advanced stage, the effect of surgical treatment is limited, and the 5-year survival rate is only 25%. In the early stages of esophageal cancer, endoscopic resection is a treatment option with good therapeutic results, and data suggest that endoscopic treatment is equivalent to surgery in terms of overall and cancer-specific survival, with lower surgical morbidity and mortality (Naveed and Kubiliun, 2018). For patients with advanced esophageal cancer, the main purpose of treatment is to relieve symptoms and prolong the survival time of patients. Concurrent chemoradiotherapy may achieve good curative effect (Herskovic et al., 2012). MO-I-500, as a kind of particular FTO inhibitor, can inhibit the aggressive inflammatory breast cancer cell line SUM149-MA or cell colony formation of survival (Singh et al., 2016). IGF2BP3 stabilizes COX6B2 to increase oxidative phosphorylation and drive resistance to EGFR inhibitors in lung cancer, providing a therapeutic strategy to overcome acquired resistance by targeting metabolic shifts (Lin et al., 2023). Yankova et al. (2021) found that the METTL3 inhibitor STM2457 reduced the growth of acute myeloid leukemia AML and increased differentiation and apoptosis. Sun et al. (2023a) also found that METTL3 promoted chemotherapy resistance in small cell lung cancer by positively regulating mitophagy, while STM2457 could reverse chemotherapy resistance in small cell lung cancer. The anti-HIV drug Elvitegravir has been shown to enhance the ubiquitination and subsequent degradation of METTL3 by facilitating its interaction with the E3 ubiquitin ligase STUB1. This mechanism highlights the drug’s significant inhibitory effect on the invasion and metastasis of EC (Liao et al., 2022). Despite these promising findings, the development of inhibitors targeting m6A regulatory proteins is still in its early stages. Current inhibitors often face challenges such as low activity and poor specificity. Most m6A regulatory protein inhibitors are primarily employed in preclinical studies, with no reported applications in EC patients to date. This underscores the need for further research to develop more effective and targeted therapies based on m6A regulation in esophageal cancer. An overview picture summarizing the therapeutic targeting of m6A is shown in Figure 4.

## 6 Regulation of cellular signaling pathways

Recent studies have shown that m6A regulation extends beyond the modulation of mRNA stability, influencing key cellular signaling pathways involved in cell proliferation, migration, and immune response. These pathways include the PI3K/Akt, Wnt/β-catenin, and JAK/STAT pathways and so on, all of which are critical in tumorigenesis and metastasis.

Previous studies have established a strong correlation between the activation of the JAK-STAT signaling pathway and various diseases (Yue et al., 2020),including non-small lung cancer (Prabhu et al., 2021), breast cancer (Chen J. et al., 2021) and ovarian cancer (Gao et al., 2022). Sun et al. (2023b) demonstrated that the m6A methyltransferase METTL3 enhances the translation of critical oncogenes, such as JAK1 and STAT3, which play a pivotal role in the signaling cascade driving colorectal cancer progression. By promoting m6A modification, METTL3 facilitates the activation of the JAK/STAT signaling pathway, thereby contributing to the oncogenic transformation of colorectal cancer cells.

The PI3K/AKT signaling pathway serves as a crucial regulatory mechanism for cell proliferation and metabolism, and it is intricately linked to tumorigenesis and development. Liu X. et al. (2021) indicates that METTL14 is the main regulator for the abnormal m6A modification in GC and METTL14 suppresses GC cell progression and aggression by deactivating the PI3K/AKT/mTOR pathway and the EMT pathway as a tumor suppressor. Chronic infections with the hepatitis B virus (HBV) constitute a prominent cause of liver cirrhosis and hepatocellular carcinoma (HCC). Studies has unveiled a notable finding: HBV infection induces a substantial elevation in the m6A modification of Foxp4 mRNA, which in turn enhances the stability of this mRNA and leads to a subsequent surge in Foxp4 mRNA levels. Furthermore, researchers also demonstrated that HBV gene expression activates the PI3K/AKT pathway in HCC cells by modulating the stability of Foxp4 mRNA. This study offers profound insights into the fundamental mechanisms underlying HBV infection and its potential ramifications for cancer progression (Wang et al., 2024).

Osteoarthritis (OA) is the most common form of arthritis, An et al. (2024) revealed that WTAP-dependent m6A modification of RNA activates the Wnt/β-catenin pathway and promotes OA progression by regulating FRZB mRNA post-transcriptionally, suggesting a potential therapeutic strategy for OA. Ma et al. (2024) identified and characterized PSEN1 as a novel target regulated by m6A modification during craniofacial development. Mechanistically, METTL3-mediated m6A modification controls the expression of PSEN1 by modulating its mRNA stability in a YTHDF1-dependent manner. Subsequently, PSEN1 regulates Wnt/β-catenin signaling through binding to β-catenin, thereby influencing craniofacial developmental processes via METTL3-mediated m6A modification.

## 7 Role of m5c in esophageal cancer

Previous studies have found that the expression of NSUN2 protein and mRNA is elevated in esophageal cancer tissues, and the expression of NSUN2 is higher with later tumor staging, indicating that high expression of NUSUN2 is closely related to poor prognosis in patients (Su et al., 2021; Okamoto et al., 2012). Li et al. (2016) found that TET1 expression is downregulated in esophageal cancer Shi et al. (2016) found that compared to paired non tumor tissues, the expression of TET2 and TET3 was significantly reduced in tumor tissues. TET2 is regulated by epigenetics in esophageal cancer tissue and mutations occur in nearly 6% of Japanese esophageal cancer patients (Sawada et al., 2016). According to the Japanese study, TET2 mutation can be considered as a negative prognostic indicator for the survival of esophageal cancer patients. However, Xu et al. (2020) found that the expression of TET3 was elevated and negatively correlated with patient survival rate. Through screening and experimental verification of public databases, Su et al. (2021) found a positive correlation between NSUN2 and E2F1 expression, demonstrating that E2F1 acts as a transcription factor that upregulates NSUN2 in esophageal cancer. This regulation was evidenced by significant downregulation of NSUN2 at both mRNA and protein levels following the silencing of E2F1. Niu et al. (2022) further investigated the genetic aspects of NSUN2 expression, identifying rs10076470 G to A mutations in the NSUN2 gene in certain ESCC patients. These mutations led to the formation of cis eQTLs, which bind to STAT1 and transcription factors, thereby promoting the expression of NSUN2. This highlights the role of genetic variations in influencing NSUN2 levels and potentially impacting esophageal cancer progression. The TET1 promoter and exon 1 region have typical methylated CpG islands, and carcinogen-induced CpG methylation can inhibit TET1 expression in esophageal cells (Li et al., 2016). TET2 expression is regulated negatively by the microRNA-196a/UHRF2 axis, indicating a complex interplay between microRNAs and TET family members in esophageal cancer (Hu et al., 2022). Xu et al. (2020) found that TET3 is upregulated via the TLR4/p38/ERK-MAPK pathway in response to lipopolysaccharide stimulation, and this upregulation is negatively correlated with patient survival. This suggests that TET3 may play a role in tumor progression. NSUN2 enhances the expression of various cancer-related genes through mRNA m5C methylation, which contributes to radiochemotherapy resistance in patients (Niu et al., 2022). It mediates the m5C modification of GRB2, promoting its interaction with LIN28B, a key m5C mediator alongside YBX1 and ALYREF. The binding of LIN28B stabilizes GRB2 mRNA, leading to the activation of important signaling pathways like PI3K/AKT and ERK/MAPK, further influencing tumor growth and survival (Su et al., 2021). Li et al. (2018) identified lncRNA NMR as a methylation target of NSUN2, playing various roles in esophageal cancer. m 5C-methylated NMR can increase chemoresistance in ESCC cells and bind to the chromatin regulator BPTF to mediate tumor progression. Interestingly, methylated NMR may also inhibit the methylation of certain mRNAs associated with cell migration and invasion, including LAMB1, PLOD3, HSPG2, and COL4A5. YBX1 interacts with the 3′UTR of c-Myc mRNA, aided by the linc02042 scaffold, which enhances c-Myc mRNA stability. This stabilization promotes the proliferation and metastasis of EC (Du et al., 2020). The TET family, while not extensively discussed in terms of its function as RNA methylation regulators, has been investigated in relation to esophageal cancer. LncRNA ZNF667-AS1 activates its target genes, ZNF667 and E-cadherin, by recruiting TET1, thereby inhibiting ESCC progression (Dong et al., 2019). The suppression of TET1 demethylase is linked to low chromosomal copy-number alterations (CNAs) in HPV-positive EC (Campbell et al., 2018). Han et al. (2022) elucidated that loss of TET1 expression promotes tumor suppressor gene inactivation in tumor pathogenesis. Knockdown of TET2 enhances malignant phenotypes of ESCC by increasing cell proliferation, migration, and invasion (Sawada et al., 2016; Hu et al., 2022). Moreover, overexpression of TET3 has been reported to induce the stemness of EC cells (Xu et al., 2020).

## 8 Role of m7G in esophageal cancer

In 2022, Han et al. (2022) demonstrated that the m7G methyltransferase complex proteins METTL1 and WDR4 are significantly upregulated in ESCC tissues and are associated with adverse prognosis in ESCC. Knockdown of METTL1 or WDR4 can lead to reduced expression of m7G-modified tRNA and decrease translation of a subset of oncogenic transcripts enriched in the RPTOR/ULK1/autophagy pathway. High WBSCR22 expression predicts adverse prognosis in ESCC patients (Li C. et al., 2017). Zhao et al. (2022) developed a robust and effective prognostic marker related to m7G regulatory factors for LNCRNA, which offers superior predictive value compared to traditional clinical risk factors. Their findings revealed that, compared to normal adjacent tissues, expression levels of AC025754.2, AL4, 51165.2, and AL513550.1 significantly increase in ESCC tissues, whereas HAND2-AS1, SNHG7, SRP14-AS1, and AC007566.1 are downregulated in ESCC tissues.

## 9 m6A modification in other cancers

Beyond esophageal cancer, N6-methyladenosine (m6A) RNA methylation has been identified as a critical factor in the pathogenesis of various malignancies. In hepatocellular carcinoma (HCC), m6A regulators such as METTL3 and FTO have been demonstrated to modulate tumor progression by influencing the stability and translation efficiency of oncogenic mRNAs (Chen et al., 2018). Similarly, in glioblastoma, m6A modification regulates cancer stem cell maintenance and tumorigenesis through modulation of key signaling pathways, including Wnt/β-catenin (Cui et al., 2017). In breast cancer, m6A methylation has been linked to epithelial-mesenchymal transition (EMT) and metastasis, with METTL14 playing a critical role in suppressing tumor invasion (Zhang et al., 2016). Furthermore, in acute myeloid leukemia (AML), aberrant m6A levels contribute to dysregulated hematopoietic differentiation and leukemogenesis, emphasizing the extensive influence of m6A modification on cancer biology (Vu et al., 2017). Another research revealed that the m6A demethylase ALKBH5 was markedly downregulated in GC tissues, which was associated with poor patient prognosis (Zheng et al., 2025). Collectively, these findings highlight the pervasive and multifaceted role of m6A methylation across diverse cancer types, suggesting its potential as a universal therapeutic target.

## 10 Conclusion

There has been an increasing awareness of RNA methylation’s role in esophageal cancer in recent years, but m6A modification research is still in its infancy, posing various challenges. The purpose of this review is to discuss m6A, m5C, and m7G modifications of RNA in esophageal cancer. We begin by describing the three types of RNA methylation and their respective regulators. Subsequently, we highlight the roles and clinical significance of these RNA methylation regulators in esophageal cancer, emphasizing their potential impact on tumor progression and patient outcomes. Furthermore, we conduct a systematic review of the regulatory expression of these factors in esophageal cancer, synthesizing findings from published studies to offer a comprehensive overview of their implications in this malignancy. The purpose of this work is to better understand how RNA methylation is related to esophageal cancer, as well as to identify future research and therapeutic avenues.

There has been a growing interest in immunotherapy as a treatment strategy for esophageal cancer, highlighting the importance of targeting RNA methylation within the tumor microenvironment. To understand the molecular mechanisms behind esophageal cancer, a holistic approach must be taken that takes into account both tumor cells and the surrounding microenvironment (Lin EW. et al., 2016). Research has shown that targeting m6A modification can influence the response to immunotherapy. Similarly, Liu et al. (2021c) suggested that targeting m6A modifications could enhance the efficacy of anti-PD-1 therapy in non-small cell lung cancer. Wan et al. (2022) demonstrated that m6A modification of PD-L1 mRNA stabilizes its expression in breast cancer, thereby inhibiting immune surveillance. Moreover, several studies indicate that targeting m6A could help overcome chemoradiotherapy resistance in various malignant tumors, including esophageal cancer. Therefore, future research should prioritize exploring the roles of m6A methylation regulators in the context of ESCC tumor immunotherapy, as this could reveal new strategies for enhancing treatment responses and improving patient outcomes.

The emergence of mutations at m6A methylation sites has garnered significant attention due to their potential impact on RNA function and regulation. However, the frequency of mutations occurring at the m6A locus and their potential consequences remain largely unexplored. This phenomenon is influenced by multiple factors, such as the genomic background, the specificity of surrounding sequences, and the biological or pathological state of the cell. Once a mutation occurs, it may disrupt the normal process of m6A modification, thereby affecting gene expression regulation. Studies have reported varying mutation frequencies among m6A regulatory genes. For instance, an analysis Wang et al. (2022) revealed a mutation rate of 4.69% in m6A regulators, with YTHDC2 being notably affected. Another comprehensive study identified ZC3H13 as having the highest mutation frequency among m6A-related genes (Jiang et al., 2023). In addition, a study analyzing 184 endometrial carcinoma patients found that 12.5% exhibited mutations in m6A regulators (Sheng et al., 2023). CRISPR-Cas9 technology has emerged as a promising tool for addressing these mutations by enabling precise genome editing that includes targeted modifications to m6A-related sequences within RNA transcripts (Vaghari-Tabari et al., 2022; Shi et al., 2022). Additionally, RNA-focused technologies, such as CRISPR-Cas13, provide direct RNA editing capabilities. This enables the precise correction of m6A-related mutations in a transcript-specific manner without necessitating alterations to the genome (Abudayyeh et al., 2017; Cox et al., 2017). However, the use of CRISPR-Cas9 or Cas13 in this context must overcome challenges such as off-target effects, the transient nature of RNA editing, and the precise targeting of m6A-associated sequences. Despite these challenges, initial studies have demonstrated the feasibility of employing CRISPR-based tools to restore the functionality of mutated m6A sites, suggesting a promising avenue for therapeutic applications.

While the role of m6A modification in esophageal cancer (EC) has attracted substantial attention, significant challenges and unresolved controversies remain in this rapidly advancing field. First, the functional duality of m6A regulators presents a major challenge. For instance, ALKBH5 has been reported to function as both a tumor suppressor by inhibiting proliferation and metastasis (Xiao et al., 2021) and an oncogene by promoting cell cycle progression (Nagaki et al., 2020). This highlights context-dependent roles that may vary with tumor stage, molecular subtypes, or microenvironmental cues. Similarly, METTL3 exhibits oncogenic effects via AKT/EGR1 signaling (Hou et al., 2020), contrasting with its potential tumor-suppressive roles in other cancers. These findings suggest tissue-specific regulatory networks that warrant further investigation. Such discrepancies emphasize the necessity for standardized models and larger multi-center clinical cohorts to validate findings across diverse populations. Second, the dynamic interplay between m6A and other RNA modifications (e.g., m5C, m7G) remains incompletely understood. For example, NSUN2-mediated m5C modification synergizes with m6A to stabilize oncogenic transcripts such as GRB2 in EC (Su et al., 2021). However, the mechanisms by which these modifications co-regulate RNA metabolism or compete for shared substrates remain unclear. Additionally, the clinical significance of m6A-related mutations and their influence on therapeutic resistance remain speculative, underscoring the need for functional genomics studies to elucidate mutation-specific mechanisms. Third, translational gaps impede progress. While inhibitors targeting METTL3 (e.g., STM2457) or FTO (e.g., MO-I-500) demonstrate preclinical promise, their efficacy and toxicity in EC patients have yet to be evaluated. Furthermore, non-invasive detection of m6A markers via liquid biopsies (e.g., circ-SLC7A5) requires validation in prospective clinical trials. The integration of m6A profiling with immunotherapy response, as observed in non-small cell lung cancer (NSCLC), remains unexplored in EC despite the increasing importance of immune checkpoint inhibitors in clinical practice. Finally, emerging technologies such as single-cell m6A sequencing and CRISPR-based RNA editing tools (e.g., Cas13) hold potential for resolving spatial heterogeneity in m6A deposition and enabling precise modulation of specific epitranscriptomic targets. Addressing these challenges will not only clarify the mechanistic complexities of m6A in EC but also accelerate the development of RNA methylation-based diagnostics and therapies, bridging the gap between basic research and clinical applications.

## References

[B1] Abudayyeh O. O. Gootenberg J. S. Essletzbichler P. Han S. Joung J. Belanto J. J. (2017). Rna targeting with crispr–cas13. Nature 550 (7675), 280–284. 10.1038/nature24049 28976959 PMC5706658

[B2] Aguilo F. Zhang F. Sancho A. Fidalgo M. Di Cecilia S. Vashisht A. (2015). Coordination of M(6)a mrna methylation and gene transcription by Zfp217 regulates pluripotency and reprogramming. Cell Stem Cell 17 (6), 689–704. 10.1016/j.stem.2015.09.005 26526723 PMC4671830

[B3] An X. Wang R. Lv Z. Wu W. Sun Z. Wu R. (2024). Wtap-mediated M(6)a modification of frzb triggers the inflammatory response via the Wnt signaling pathway in osteoarthritis. Exp. Mol. Med. 56 (1), 156–167. 10.1038/s12276-023-01135-5 38172596 PMC10834961

[B4] Blot W. J. Devesa S. S. Kneller R. W. Fraumeni J. F. Jr (1991). Rising incidence of adenocarcinoma of the esophagus and gastric cardia. JAMA 265 (10), 1287–1289. 10.1001/jama.1991.03460100089030 1995976

[B5] Bray F. Laversanne M. Sung H. Ferlay J. Siegel R. L. Soerjomataram I. (2024). Global cancer statistics 2022: globocan estimates of incidence and mortality worldwide for 36 cancers in 185 countries. CA Cancer J. Clin. 74 (3), 229–263. 10.3322/caac.21834 38572751

[B6] Brown J. A. Kinzig C. G. DeGregorio S. J. Steitz J. A. (2016). Methyltransferase-like protein 16 binds the 3'-terminal triple helix of Malat1 long noncoding rna. Proc. Natl. Acad. Sci. U. S. A. 113 (49), 14013–14018. 10.1073/pnas.1614759113 27872311 PMC5150381

[B7] Buas M. F. He Q. Johnson L. G. Onstad L. Levine D. M. Thrift A. P. (2017). Germline variation in inflammation-related pathways and risk of barrett's oesophagus and oesophageal adenocarcinoma. Gut 66 (10), 1739–1747. 10.1136/gutjnl-2016-311622 27486097 PMC5296402

[B8] Campbell J. D. Yau C. Bowlby R. Liu Y. Brennan K. Fan H. (2018). Genomic, pathway network, and immunologic features distinguishing squamous carcinomas. Cell Rep. 23 (1), 194–212.e6. 10.1016/j.celrep.2018.03.063 29617660 PMC6002769

[B9] Chen J. Cheng L. Zou W. Wang R. Wang X. Chen Z. (2021d). Adamts9-As1 constrains breast cancer cell invasion and proliferation via sequestering mir-301b-3p. Front. Cell Dev. Biol. 9, 719993. 10.3389/fcell.2021.719993 34900984 PMC8652087

[B10] Chen M. Wei L. Law C. T. Tsang F. H. Shen J. Cheng C. L. (2018). Rna N6-methyladenosine methyltransferase-like 3 promotes liver cancer progression through ythdf2-dependent posttranscriptional silencing of Socs2. Hepatology 67 (6), 2254–2270. 10.1002/hep.29683 29171881

[B11] Chen P. Li S. Zhang K. Zhao R. Cui J. Zhou W. (2021c). N(6)-Methyladenosine demethylase Alkbh5 suppresses malignancy of esophageal cancer by regulating microrna biogenesis and Rai1 expression. Oncogene 40 (37), 5600–5612. 10.1038/s41388-021-01966-4 34312488

[B12] Chen W. Zhang Z. Fang X. Xiong L. Wen Y. Zhou J. (2021a). Prognostic value of the albi grade among patients with single hepatocellular carcinoma without macrovascular invasion. Med. Baltim. 100 (24), e26265. 10.1097/md.0000000000026265 PMC8213286 34128857

[B13] Chen X. Huang L. Yang T. Xu J. Zhang C. Deng Z. (2021b). Mettl3 promotes esophageal squamous cell carcinoma metastasis through enhancing Gls2 expression. Front. Oncol. 11, 667451. 10.3389/fonc.2021.667451 34094960 PMC8170325

[B14] Choe J. Lin S. Zhang W. Liu Q. Wang L. Ramirez-Moya J. (2018). Mrna circularization by mettl3-eif3h enhances translation and promotes oncogenesis. Nature 561 (7724), 556–560. 10.1038/s41586-018-0538-8 30232453 PMC6234840

[B15] Conn V. M. Hugouvieux V. Nayak A. Conos S. A. Capovilla G. Cildir G. (2017). A circrna from Sepallata3 regulates splicing of its cognate mrna through R-loop formation. Nat. Plants 3, 17053. 10.1038/nplants.2017.53 28418376

[B16] Cox D. B. T. Gootenberg J. S. Abudayyeh O. O. Franklin B. Kellner M. J. Joung J. (2017). Rna editing with crispr-cas13. Science 358 (6366), 1019–1027. 10.1126/science.aaq0180 29070703 PMC5793859

[B17] Cui Q. Shi H. Ye P. Li L. Qu Q. Sun G. (2017). M(6)a rna methylation regulates the self-renewal and tumorigenesis of glioblastoma stem cells. Cell Rep. 18 (11), 2622–2634. 10.1016/j.celrep.2017.02.059 28297667 PMC5479356

[B18] Cui Y. Zhang C. Ma S. Li Z. Wang W. Li Y. (2021). Rna M6a demethylase fto-mediated epigenetic up-regulation of Linc00022 promotes tumorigenesis in esophageal squamous cell carcinoma. J. Exp. Clin. Cancer Res. 40 (1), 294. 10.1186/s13046-021-02096-1 34544449 PMC8451109

[B19] Dong Z. Li S. Wu X. Niu Y. Liang X. Yang L. (2019). Aberrant hypermethylation-mediated downregulation of antisense lncrna znf667-as1 and its sense gene Znf667 correlate with progression and prognosis of esophageal squamous cell carcinoma. Cell Death Dis. 10 (12), 930. 10.1038/s41419-019-2171-3 31804468 PMC6895126

[B20] Du H. Zhao Y. He J. Zhang Y. Xi H. Liu M. (2016). Ythdf2 destabilizes M(6)a-containing rna through direct recruitment of the ccr4-not deadenylase complex. Nat. Commun. 7, 12626. 10.1038/ncomms12626 27558897 PMC5007331

[B21] Du J. Zhang G. Qiu H. Yu H. Yuan W. (2020). A novel positive feedback loop of Linc02042 and C-myc mediated by Ybx1 promotes tumorigenesis and metastasis in esophageal squamous cell carcinoma. Cancer Cell Int. 20, 75. 10.1186/s12935-020-1154-x 32161513 PMC7060651

[B22] Duan X. Yang L. Wang L. Liu Q. Zhang K. Liu S. (2022). M6a demethylase fto promotes tumor progression via regulation of lipid metabolism in esophageal cancer. Cell Biosci. 12 (1), 60. 10.1186/s13578-022-00798-3 35568876 PMC9107638

[B23] Gao A. H. Hu Y. R. Zhu W. P. (2022). Ifn-Γ inhibits ovarian cancer progression via socs1/jak/stat signaling pathway. Clin. Transl. Oncol. 24 (1), 57–65. 10.1007/s12094-021-02668-9 34275119

[B24] Gong P. J. Shao Y. C. Yang Y. Song W. J. He X. Zeng Y. F. (2020). Analysis of N6-methyladenosine methyltransferase reveals Mettl14 and Zc3h13 as tumor suppressor genes in breast cancer. Front. Oncol. 10, 578963. 10.3389/fonc.2020.578963 33363011 PMC7757663

[B25] Guo H. Wang B. Xu K. Nie L. Fu Y. Wang Z. (2020). M(6)a reader Hnrnpa2b1 promotes esophageal cancer progression via up-regulation of acly and Acc1. Front. Oncol. 10, 553045. 10.3389/fonc.2020.553045 33134163 PMC7550530

[B26] Guo X. F. Fang W. T. (2021). The role of neoadjuvant therapy in multimodality treatment of locally advanced esophageal squamous cell carcinoma: perspective from the Neocrtec5010 trial. Zhonghua Wai Ke Za Zhi 59 (8), 646–650. 10.3760/cma.j.cn112139-20210109-00021 34192855

[B27] Hagen Pv Hulshof M. C. C. M. Jjbv L. Steyerberg E. W. MivB H. Wijnhoven B. P. L. (2012). Preoperative chemoradiotherapy for esophageal or junctional cancer. N. Engl. J. Med. 366 (22), 2074–2084. 10.1056/NEJMoa1112088 22646630

[B28] Han H. Yang C. Ma J. Zhang S. Zheng S. Ling R. (2022). N(7)-Methylguanosine trna modification promotes esophageal squamous cell carcinoma tumorigenesis via the rptor/ulk1/autophagy Axis. Nat. Commun. 13 (1), 1478. 10.1038/s41467-022-29125-7 35304469 PMC8933395

[B29] Hansen T. B. Jensen T. I. Clausen B. H. Bramsen J. B. Finsen B. Damgaard C. K. (2013). Natural rna circles function as efficient microrna sponges. Nature 495 (7441), 384–388. 10.1038/nature11993 23446346

[B30] He C. Teng X. Wang L. Ni M. Zhu L. Liu J. (2023). The implications of N6-methyladenosine (M6a) modification in esophageal carcinoma. Mol. Biol. Rep. 50 (10), 8691–8703. 10.1007/s11033-023-08575-2 37598390 PMC10520198

[B31] Herskovic A. Russell W. Liptay M. Fidler M. J. Al-Sarraf M. (2012). Esophageal carcinoma advances in treatment results for locally advanced disease: review. Ann. Oncol. 23 (5), 1095–1103. 10.1093/annonc/mdr433 22003242

[B32] Hou H. Zhao H. Yu X. Cong P. Zhou Y. Jiang Y. (2020). Mettl3 promotes the proliferation and invasion of esophageal cancer cells partly through akt signaling pathway. Pathol. Res. Pract. 216 (9), 153087. 10.1016/j.prp.2020.153087 32825955

[B33] Hsu P. J. Zhu Y. Ma H. Guo Y. Shi X. Liu Y. (2017). Ythdc2 is an N(6)-methyladenosine binding protein that regulates mammalian spermatogenesis. Cell Res. 27 (9), 1115–1127. 10.1038/cr.2017.99 28809393 PMC5587856

[B34] Hu C. M. Peng J. Lv L. Wang X. H. Huo J. R. Liu D. L. (2022). Mir-196a promotes the proliferation and migration of esophageal cancer via the uhrf2/tet2 Axis. Mol. Cell Biochem. 477 (2), 537–547. 10.1007/s11010-021-04301-3 34826027

[B35] Huang F. L. Yu S. J. (2018). Esophageal cancer: risk factors, genetic association, and treatment. Asian J. Surg. 41 (3), 210–215. 10.1016/j.asjsur.2016.10.005 27986415

[B36] Huang H. Weng H. Sun W. Qin X. Shi H. Wu H. (2018). Recognition of rna N(6)-methyladenosine by Igf2bp proteins enhances mrna stability and translation. Nat. Cell Biol. 20 (3), 285–295. 10.1038/s41556-018-0045-z 29476152 PMC5826585

[B37] Huang Q. Mo J. Liao Z. Chen X. Zhang B. (2022). The rna M(6)a writer wtap in diseases: structure, roles, and mechanisms. Cell Death Dis. 13 (10), 852. 10.1038/s41419-022-05268-9 36207306 PMC9546849

[B38] Jackson R. J. Hellen C. U. Pestova T. V. (2010). The mechanism of eukaryotic translation initiation and principles of its regulation. Nat. Rev. Mol. Cell Biol. 11 (2), 113–127. 10.1038/nrm2838 20094052 PMC4461372

[B39] Jiang T. Xing L. Zhao L. Ye Z. Yu D. Lin S. (2023). Comprehensive analysis of M6a related gene mutation characteristics and prognosis in colorectal cancer. BMC Med. Genomics 16 (1), 105. 10.1186/s12920-023-01509-8 37194014 PMC10186803

[B40] Jiang X. Liu B. Nie Z. Duan L. Xiong Q. Jin Z. (2021). The role of M6a modification in the biological functions and diseases. Signal Transduct. Target Ther. 6 (1), 74. 10.1038/s41392-020-00450-x 33611339 PMC7897327

[B41] Kane S. E. Beemon K. (1985). Precise localization of M6a in rous sarcoma virus rna reveals clustering of methylation sites: implications for rna processing. Mol. Cell Biol. 5 (9), 2298–2306. 10.1128/mcb.5.9.2298 3016525 PMC366956

[B42] Kit O. I. Vodolazhskiy D. I. Kolesnikov E. N. Timoshkina N. N. (2016). Epigenetic markers of esophageal cancer: DNA methylation. Biomed. Khim 62 (5), 520–526. 10.18097/pbmc20166205520 27797326

[B43] Knuckles P. Lence T. Haussmann I. U. Jacob D. Kreim N. Carl S. H. (2018). Zc3h13/Flacc is required for adenosine methylation by bridging the mrna-binding factor rbm15/spenito to the M(6)a machinery component wtap/fl(2)D. Genes Dev. 32 (5-6), 415–429. 10.1101/gad.309146.117 29535189 PMC5900714

[B44] Kong F. Zou H. Liu X. He J. Zheng Y. Xiong L. (2020). Mir-7112-3p targets perk to regulate the endoplasmic reticulum stress pathway and apoptosis induced by photodynamic therapy in colorectal cancer cx-1 cells. Photodiagnosis Photodyn. Ther. 29, 101663. 10.1016/j.pdpdt.2020.101663 31945549

[B45] Lan T. Li H. Zhang D. Xu L. Liu H. Hao X. (2019). Kiaa1429 contributes to liver cancer progression through N6-methyladenosine-dependent post-transcriptional modification of Gata3. Mol. Cancer 18 (1), 186. 10.1186/s12943-019-1106-z 31856849 PMC6921542

[B46] Leng X. He W. Yang H. Chen Y. Zhu C. Fang W. (2021). Prognostic impact of postoperative lymph node metastases after neoadjuvant chemoradiotherapy for locally advanced squamous cell carcinoma of esophagus: from the results of Neocrtec5010, a randomized multicenter study. Ann. Surg. 274 (6), e1022–e1029. 10.1097/sla.0000000000003727 31855875

[B47] Lesbirel S. Viphakone N. Parker M. Parker J. Heath C. Sudbery I. (2018). The M(6)a-methylase complex recruits trex and regulates mrna export. Sci. Rep. 8 (1), 13827. 10.1038/s41598-018-32310-8 30218090 PMC6138711

[B48] Li A. Chen Y. S. Ping X. L. Yang X. Xiao W. Yang Y. (2017b). Cytoplasmic M(6)a reader Ythdf3 promotes mrna translation. Cell Res. 27 (3), 444–447. 10.1038/cr.2017.10 28106076 PMC5339832

[B49] Li C. Wang Q. Ma J. Shi S. Chen X. Yang H. (2017c). Integrative pathway analysis of genes and metabolites reveals metabolism abnormal subpathway regions and modules in esophageal squamous cell carcinoma. Molecules 22 (10), 1599. 10.3390/molecules22101599 28937628 PMC6151487

[B50] Li J. Liu H. Dong S. Zhang Y. Li X. Wang J. (2021a). Alkbh5 is lowly expressed in esophageal squamous cell carcinoma and inhibits the malignant proliferation and invasion of tumor cells. Comput. Math. Methods Med. 2021, 1001446. 10.1155/2021/1001446 34876920 PMC8645391

[B51] Li K. Chen J. Lou X. Li Y. Qian B. Xu D. (2021b). Hnrnpa2b1 affects the prognosis of esophageal cancer by regulating the mir-17-92 cluster. Front. Cell Dev. Biol. 9, 658642. 10.3389/fcell.2021.658642 34277606 PMC8278577

[B52] Li L. Li C. Mao H. Du Z. Chan W. Y. Murray P. (2016). Epigenetic inactivation of the cpg demethylase Tet1 as a DNA methylation feedback loop in human cancers. Sci. Rep. 6, 26591. 10.1038/srep26591 27225590 PMC4880909

[B53] Li L. Xie R. Wei Q. (2021c). Network analysis of mirna targeting m6a-related genes in patients with esophageal cancer. PeerJ 9, e11893. 10.7717/peerj.11893 34395102 PMC8325912

[B54] Li T. Hu P. S. Zuo Z. Lin J. F. Li X. Wu Q. N. (2019). Mettl3 facilitates tumor progression via an M(6)a-igf2bp2-dependent mechanism in colorectal carcinoma. Mol. Cancer 18 (1), 112. 10.1186/s12943-019-1038-7 31230592 PMC6589893

[B55] Li Y. Li J. Luo M. Zhou C. Shi X. Yang W. (2018). Novel long noncoding rna nmr promotes tumor progression via Nsun2 and bptf in esophageal squamous cell carcinoma. Cancer Lett. 430, 57–66. 10.1016/j.canlet.2018.05.013 29763634

[B56] Li Y. Su R. Deng X. Chen Y. Chen J. (2022). Fto in cancer: functions, molecular mechanisms, and therapeutic implications. Trends Cancer 8 (7), 598–614. 10.1016/j.trecan.2022.02.010 35346615

[B57] Li Z. Weng H. Su R. Weng X. Zuo Z. Li C. (2017a). Fto plays an oncogenic role in acute myeloid leukemia as a N(6)-methyladenosine rna demethylase. Cancer Cell 31 (1), 127–141. 10.1016/j.ccell.2016.11.017 28017614 PMC5234852

[B58] Liang X. Zhang Z. Wang L. Zhang S. Ren L. Li S. (2021). Mechanism of methyltransferase like 3 in epithelial-mesenchymal transition process, invasion, and metastasis in esophageal cancer. Bioengineered 12 (2), 10023–10036. 10.1080/21655979.2021.1994721 34666602 PMC8810097

[B59] Liao L. He Y. Li S. J. Zhang G. G. Yu W. Yang J. (2022). Anti-hiv drug Elvitegravir suppresses cancer metastasis via increased proteasomal degradation of M6a methyltransferase Mettl3. Cancer Res. 82 (13), 2444–2457. 10.1158/0008-5472.Can-21-4124 35507004

[B60] Lin D.-C. Hao J.-J. Nagata Y. Xu L. Shang L. Meng X. (2014). Genomic and molecular characterization of esophageal squamous cell carcinoma. Nat. Genet. 46 (5), 467–473. 10.1038/ng.2935 24686850 PMC4070589

[B61] Lin E. W. Karakasheva T. A. Hicks P. D. Bass A. J. Rustgi A. K. (2016b). The tumor microenvironment in esophageal cancer. Oncogene 35 (41), 5337–5349. 10.1038/onc.2016.34 26923327 PMC5003768

[B62] Lin S. Choe J. Du P. Triboulet R. Gregory R. I. (2016a). The M(6)a methyltransferase Mettl3 promotes translation in human cancer cells. Mol. Cell 62 (3), 335–345. 10.1016/j.molcel.2016.03.021 27117702 PMC4860043

[B63] Lin Z. Li J. Zhang J. Feng W. Lu J. Ma X. (2023). Metabolic reprogramming driven by Igf2bp3 promotes acquired resistance to egfr inhibitors in non-small cell lung cancer. Cancer Res. 83 (13), 2187–2207. 10.1158/0008-5472.Can-22-3059 37061993

[B64] Liu J. Yue Y. Han D. Wang X. Fu Y. Zhang L. (2014). A mettl3-mettl14 complex mediates mammalian nuclear rna N6-adenosine methylation. Nat. Chem. Biol. 10 (2), 93–95. 10.1038/nchembio.1432 24316715 PMC3911877

[B65] Liu N. Dai Q. Zheng G. He C. Parisien M. Pan T. (2015). N(6)-Methyladenosine-Dependent rna structural switches regulate rna-protein interactions. Nature 518 (7540), 560–564. 10.1038/nature14234 25719671 PMC4355918

[B66] Liu N. Zhou K. I. Parisien M. Dai Q. Diatchenko L. Pan T. (2017). N6-Methyladenosine alters rna structure to regulate binding of a low-complexity protein. Nucleic Acids Res. 45 (10), 6051–6063. 10.1093/nar/gkx141 28334903 PMC5449601

[B67] Liu S. Huang M. Chen Z. Chen J. Chao Q. Yin X. (2020a). Fto promotes cell proliferation and migration in esophageal squamous cell carcinoma through up-regulation of Mmp13. Exp. Cell Res. 389 (1), 111894. 10.1016/j.yexcr.2020.111894 32035950

[B68] Liu X. Xiao M. Zhang L. Li L. Zhu G. Shen E. (2021b). The M6a methyltransferase Mettl14 inhibits the proliferation, migration, and invasion of gastric cancer by regulating the pi3k/akt/mtor signaling pathway. J. Clin. Lab. Anal. 35 (3), e23655. 10.1002/jcla.23655 33314339 PMC7957981

[B69] Liu X. S. Yuan L. L. Gao Y. Zhou L. M. Yang J. W. Pei Z. J. (2020b). Overexpression of Mettl3 associated with the metabolic status on (18)F-fdg pet/ct in patients with esophageal carcinoma. J. Cancer 11 (16), 4851–4860. 10.7150/jca.44754 32626532 PMC7330681

[B70] Liu Y. Shi S. L. (2021). The roles of hnrnp A2/B1 in rna biology and disease. Wiley Interdiscip. Rev. RNA 12 (2), e1612. 10.1002/wrna.1612 32588964

[B71] Liu Z. Wang T. She Y. Wu K. Gu S. Li L. (2021c). N(6)-Methyladenosine-Modified Circigf2bp3 inhibits Cd8(+) T-cell responses to facilitate tumor immune evasion by promoting the deubiquitination of Pd-L1 in non-small cell lung cancer. Mol. Cancer 20 (1), 105. 10.1186/s12943-021-01398-4 34416901 PMC8377850

[B72] Liu Z. Wu K. Gu S. Wang W. Xie S. Lu T. (2021a). A methyltransferase-like 14/mir-99a-5p/tribble 2 positive feedback circuit promotes cancer stem cell persistence and radioresistance via histone deacetylase 2-mediated epigenetic modulation in esophageal squamous cell carcinoma. Clin. Transl. Med. 11 (9), e545. 10.1002/ctm2.545 34586732 PMC8441142

[B73] Ma H. Wang X. Cai J. Dai Q. Natchiar S. K. Lv R. (2019). N(6-)Methyladenosine methyltransferase Zcchc4 mediates ribosomal rna methylation. Nat. Chem. Biol. 15 (1), 88–94. 10.1038/s41589-018-0184-3 30531910 PMC6463480

[B74] Ma L. Zhou X. Yao S. Zhang X. Mao J. Vona B. (2024). Mettl3-Dependent M(6)a modification of Psen1 mrna regulates craniofacial development through the wnt/Β-catenin signaling pathway. Cell Death Dis. 15 (3), 229. 10.1038/s41419-024-06606-9 38509077 PMC10954657

[B75] Muthusamy S. (2020). M(6)a mrna methylation: a pleiotropic regulator of cancer. Gene 736, 144415. 10.1016/j.gene.2020.144415 32006598

[B76] Nagaki Y. Motoyama S. Yamaguchi T. Hoshizaki M. Sato Y. Sato T. (2020). M(6) a demethylase Alkbh5 promotes proliferation of esophageal squamous cell carcinoma associated with poor prognosis. Genes cells 25 (8), 547–561. 10.1111/gtc.12792 32449584

[B77] Naveed M. Kubiliun N. (2018). Endoscopic treatment of early-stage esophageal cancer. Curr. Oncol. Rep. 20 (9), 71. 10.1007/s11912-018-0713-y 30058019

[B78] Niu X. Peng L. Liu W. Miao C. Chen X. Chu J. (2022). A cis-eqtl in Nsun2 promotes esophageal squamous-cell carcinoma progression and radiochemotherapy resistance by mrna-M(5)C methylation. Signal Transduct. Target Ther. 7 (1), 267. 10.1038/s41392-022-01063-2 35934711 PMC9357702

[B79] Okamoto M. Hirata S. Sato S. Koga S. Fujii M. Qi G. (2012). Frequent increased gene copy number and high protein expression of trna (Cytosine-5-)-Methyltransferase (Nsun2) in human cancers. DNA Cell Biol. 31 (5), 660–671. 10.1089/dna.2011.1446 22136356

[B80] Parashar N. C. Parashar G. Nayyar H. Sandhir R. (2018). N(6)-Adenine DNA methylation demystified in eukaryotic genome: from biology to pathology. Biochimie 144, 56–62. 10.1016/j.biochi.2017.10.014 29074394

[B81] Pendleton K. E. Chen B. Liu K. Hunter O. V. Xie Y. Tu B. P. (2017). The U6 snrna M(6)a methyltransferase Mettl16 regulates sam synthetase intron retention. Cell 169 (5), 824–835. 10.1016/j.cell.2017.05.003 28525753 PMC5502809

[B82] Pennathur A. Gibson M. K. Jobe B. A. Luketich J. D. (2013). Oesophageal carcinoma. Lancet 381 (9864), 400–412. 10.1016/s0140-6736(12)60643-6 23374478

[B83] Pestova T. V. Kolupaeva V. G. (2002). The roles of individual eukaryotic translation initiation factors in ribosomal scanning and initiation codon selection. Genes Dev. 16 (22), 2906–2922. 10.1101/gad.1020902 12435632 PMC187480

[B84] Pestova T. V. Kolupaeva V. G. Lomakin I. B. Pilipenko E. V. Shatsky I. N. Agol V. I. (2001). Molecular mechanisms of translation initiation in eukaryotes. Proc. Natl. Acad. Sci. U. S. A. 98 (13), 7029–7036. 10.1073/pnas.111145798 11416183 PMC34618

[B85] Prabhu K. S. Bhat A. A. Siveen K. S. Kuttikrishnan S. Raza S. S. Raheed T. (2021). Sanguinarine mediated apoptosis in non-small cell lung cancer via generation of reactive oxygen species and suppression of jak/stat pathway. Biomed. Pharmacother. 144, 112358. 10.1016/j.biopha.2021.112358 34794241

[B86] Rong B. Zhang Q. Wan J. Xing S. Dai R. Li Y. (2020). Ribosome 18s M(6)a methyltransferase Mettl5 promotes translation initiation and breast cancer cell growth. Cell Rep. 33 (12), 108544. 10.1016/j.celrep.2020.108544 33357433

[B87] Roundtree I. A. Luo G. Z. Zhang Z. Wang X. Zhou T. Cui Y. (2017). Ythdc1 mediates nuclear export of N(6)-methyladenosine methylated mrnas. Elife 6, e31311. 10.7554/eLife.31311 28984244 PMC5648532

[B88] Satterwhite E. R. Mansfield K. D. (2022). Rna methyltransferase Mettl16: targets and function. Wiley Interdiscip. Rev. RNA 13 (2), e1681. 10.1002/wrna.1681 34227247 PMC9286414

[B89] Sawada G. Niida A. Uchi R. Hirata H. Shimamura T. Suzuki Y. (2016). Genomic landscape of esophageal squamous cell carcinoma in a Japanese population. Gastroenterology 150 (5), 1171–1182. 10.1053/j.gastro.2016.01.035 26873401

[B90] Shapiro J. van Lanschot J. J. B. Hulshof MCCM van Hagen P. van Berge Henegouwen M. I. Wijnhoven B. P. L. (2015). Neoadjuvant chemoradiotherapy plus surgery versus surgery alone for oesophageal or junctional cancer (cross): long-term results of a randomised controlled trial. Lancet Oncol. 16 (9), 1090–1098. 10.1016/S1470-2045(15)00040-6 26254683

[B91] Sheng G. Wang T. Gao Y. Wu H. Wu J. (2023). M6a regulator-mediated methylation modification patterns and tumor microenvironment immune infiltration with prognostic analysis in esophageal cancer. Sci. Rep. 13 (1), 19670. 10.1038/s41598-023-46729-1 37952076 PMC10640615

[B92] Shi H. Wang X. Lu Z. Zhao B. S. Ma H. Hsu P. J. (2017). Ythdf3 facilitates translation and decay of N(6)-methyladenosine-modified rna. Cell Res. 27 (3), 315–328. 10.1038/cr.2017.15 28106072 PMC5339834

[B93] Shi H. Xu Y. Tian N. Yang M. Liang F.-S. (2022). Inducible and reversible rna N6-methyladenosine editing. Nat. Commun. 13 (1), 1958. 10.1038/s41467-022-29665-y 35414049 PMC9005610

[B94] Shi X. Yu Y. Luo M. Zhang Z. Shi S. Feng X. (2016). Loss of 5-hydroxymethylcytosine is an independent unfavorable prognostic factor for esophageal squamous cell carcinoma. PLoS One 11 (4), e0153100. 10.1371/journal.pone.0153100 27050164 PMC4822830

[B95] Singh B. Kinne H. E. Milligan R. D. Washburn L. J. Olsen M. Lucci A. (2016). Important role of fto in the survival of rare panresistant triple-negative inflammatory breast cancer cells facing a severe metabolic challenge. PLoS One 11 (7), e0159072. 10.1371/journal.pone.0159072 27390851 PMC4938613

[B96] Sokabe M. Fraser C. S. (2014). Human eukaryotic initiation factor 2 (Eif2)-Gtp-Met-Trnai ternary complex and Eif3 stabilize the 43 S preinitiation complex. J. Biol. Chem. 289 (46), 31827–31836. 10.1074/jbc.M114.602870 25246524 PMC4231660

[B97] Su J. Wu G. Ye Y. Zhang J. Zeng L. Huang X. (2021). Nsun2-Mediated rna 5-methylcytosine promotes esophageal squamous cell carcinoma progression via lin28b-dependent Grb2 mrna stabilization. Oncogene 40 (39), 5814–5828. 10.1038/s41388-021-01978-0 34345012 PMC8484015

[B98] Sun Y. Gong W. Zhang S. (2023b). Mettl3 promotes colorectal cancer progression through activating jak1/stat3 signaling pathway. Cell Death Dis. 14 (11), 765. 10.1038/s41419-023-06287-w 38001065 PMC10673931

[B99] Sun Y. Shen W. Hu S. Lyu Q. Wang Q. Wei T. (2023a). Mettl3 promotes chemoresistance in small cell lung cancer by inducing mitophagy. J. Exp. Clin. Cancer Res. 42 (1), 65. 10.1186/s13046-023-02638-9 36932427 PMC10022264

[B100] Sung H. Ferlay J. Siegel R. L. Laversanne M. Soerjomataram I. Jemal A. (2021). Global cancer statistics 2020: globocan estimates of incidence and mortality worldwide for 36 cancers in 185 countries. CA Cancer J. Clin. 71 (3), 209–249. 10.3322/caac.21660 33538338

[B101] Thomson D. W. Dinger M. E. (2016). Endogenous microrna sponges: evidence and controversy. Nat. Rev. Genet. 17 (5), 272–283. 10.1038/nrg.2016.20 27040487

[B102] Vaghari-Tabari M. Hassanpour P. Sadeghsoltani F. Malakoti F. Alemi F. Qujeq D. (2022). Crispr/Cas9 gene editing: a new approach for overcoming drug resistance in cancer. Cell Mol. Biol. Lett. 27 (1), 49. 10.1186/s11658-022-00348-2 35715750 PMC9204876

[B103] van Tran N. Ernst F. G. M. Hawley B. R. Zorbas C. Ulryck N. Hackert P. (2019). The human 18s rrna M6a methyltransferase Mettl5 is stabilized by Trmt112. Nucleic Acids Res. 47 (15), 7719–7733. 10.1093/nar/gkz619 31328227 PMC6735865

[B104] Vu L. P. Pickering B. F. Cheng Y. Zaccara S. Nguyen D. Minuesa G. (2017). The N(6)-methyladenosine (M(6)a)-forming enzyme Mettl3 controls myeloid differentiation of normal hematopoietic and leukemia cells. Nat. Med. 23 (11), 1369–1376. 10.1038/nm.4416 28920958 PMC5677536

[B105] Wan W. Ao X. Chen Q. Yu Y. Ao L. Xing W. (2022). Mettl3/Igf2bp3 Axis inhibits tumor immune surveillance by upregulating N(6)-methyladenosine modification of Pd-L1 mrna in breast cancer. Mol. Cancer 21 (1), 60. 10.1186/s12943-021-01447-y 35197058 PMC8864846

[B106] Wang P. Doxtader K. A. Nam Y. (2016). Structural basis for cooperative function of Mettl3 and Mettl14 methyltransferases. Mol. Cell 63 (2), 306–317. 10.1016/j.molcel.2016.05.041 27373337 PMC4958592

[B107] Wang Q. Liu H. Liu Z. Yang L. Zhou J. Cao X. (2020). Circ-Slc7a5, a potential prognostic circulating biomarker for detection of escc. Cancer Genet. 240, 33–39. 10.1016/j.cancergen.2019.11.001 31726270

[B108] Wang T. T. Ji Y. M. Zhang Q. Liang B. Fan T. T. Ye X. (2024). Mettl14 induced N(6)-methyladenosine modification of Foxp4 mrna in hbv-hcc. J. Cancer 15 (19), 6232–6238. 10.7150/jca.101385 39513116 PMC11540497

[B109] Wang W. Shao F. Yang X. Wang J. Zhu R. Yang Y. (2021b). Mettl3 promotes tumour development by decreasing apc expression mediated by apc mrna N(6)-methyladenosine-dependent ythdf binding. Nat. Commun. 12 (1), 3803. 10.1038/s41467-021-23501-5 34155197 PMC8217513

[B110] Wang X. Lu Z. Gomez A. Hon G. C. Yue Y. Han D. (2014). N6-Methyladenosine-Dependent regulation of messenger rna stability. Nature 505 (7481), 117–120. 10.1038/nature12730 24284625 PMC3877715

[B111] Wang X. Tian L. Li Y. Wang J. Yan B. Yang L. (2021a). Rbm15 facilitates laryngeal squamous cell carcinoma progression by regulating Tmbim6 stability through Igf2bp3 dependent. J. Exp. Clin. Cancer Res. 40 (1), 80. 10.1186/s13046-021-01871-4 33637103 PMC7912894

[B112] Wang X. Zhao B. S. Roundtree I. A. Lu Z. Han D. Ma H. (2015). N(6)-Methyladenosine modulates messenger rna translation efficiency. Cell 161 (6), 1388–1399. 10.1016/j.cell.2015.05.014 26046440 PMC4825696

[B113] Wang Z. Zhang M. Seery S. Zheng G. Wang W. Zhao Y. (2022). Construction and validation of an M6a rna methylation regulator prognostic model for early-stage clear cell renal cell carcinoma. Oncol. Lett. 24 (2), 250. 10.3892/ol.2022.13370 35761938 PMC9214704

[B114] Warda A. S. Kretschmer J. Hackert P. Lenz C. Urlaub H. Höbartner C. (2017). Human Mettl16 is a N(6)-methyladenosine (M(6)a) methyltransferase that targets pre-mrnas and various non-coding rnas. EMBO Rep. 18 (11), 2004–2014. 10.15252/embr.201744940 29051200 PMC5666602

[B115] Watanabe M. Otake R. Kozuki R. Toihata T. Takahashi K. Okamura A. (2020). Recent progress in multidisciplinary treatment for patients with esophageal cancer. Surg. Today 50 (1), 12–20. 10.1007/s00595-019-01878-7 31535225 PMC6952324

[B116] Wojtas M. N. Pandey R. R. Mendel M. Homolka D. Sachidanandam R. Pillai R. S. (2017). Regulation of M(6)a transcripts by the 3'→5' rna helicase Ythdc2 is essential for a successful meiotic program in the mammalian germline. Mol. Cell 68 (2), 374–387. 10.1016/j.molcel.2017.09.021 29033321

[B117] Wu S. Zhang L. Deng J. Guo B. Li F. Wang Y. (2020). A novel micropeptide encoded by Y-linked Linc00278 links cigarette smoking and Ar signaling in male esophageal squamous cell carcinoma. Cancer Res. 80 (13), 2790–2803. 10.1158/0008-5472.Can-19-3440 32169859

[B118] Wu X. Fan Y. Liu Y. Shen B. Lu H. Ma H. (2021). Long non-coding rna Ccat2 promotes the development of esophageal squamous cell carcinoma by inhibiting mir-200b to upregulate the igf2bp2/tk1 Axis. Front. Oncol. 11, 680642. 10.3389/fonc.2021.680642 34386421 PMC8353391

[B119] Xia T. L. Yan S. M. Yuan L. Zeng M. S. (2020). Upregulation of Mettl3 expression predicts poor prognosis in patients with esophageal squamous cell carcinoma. Cancer Manag. Res. 12, 5729–5737. 10.2147/cmar.S245019 32765076 PMC7367742

[B120] Xiao D. Fang T. X. Lei Y. Xiao S. J. Xia J. W. Lin T. Y. (2021). M(6)a demethylase Alkbh5 suppression contributes to esophageal squamous cell carcinoma progression. Aging (Albany NY) 13 (17), 21497–21512. 10.18632/aging.203490 34491904 PMC8457604

[B121] Xiao W. Adhikari S. Dahal U. Chen Y. S. Hao Y. J. Sun B. F. (2016). Nuclear M(6)a reader Ythdc1 regulates mrna splicing. Mol. Cell 61 (4), 507–519. 10.1016/j.molcel.2016.01.012 26876937

[B122] Xu C. Wang X. Liu K. Roundtree I. A. Tempel W. Li Y. (2014). Structural basis for selective binding of M6a rna by the Ythdc1 yth domain. Nat. Chem. Biol. 10 (11), 927–929. 10.1038/nchembio.1654 25242552

[B123] Xu F. Liu Z. Liu R. Lu C. Wang L. Mao W. (2020). Epigenetic induction of tumor stemness via the lipopolysaccharide-tet3-hoxb2 signaling Axis in esophageal squamous cell carcinoma. Cell Commun. Signal 18 (1), 17. 10.1186/s12964-020-0510-8 32014008 PMC6998358

[B124] Xue J. Xiao P. Yu X. Zhang X. (2021). A positive feedback loop between alkb homolog 5 and mir-193a-3p promotes growth and metastasis in esophageal squamous cell carcinoma. Hum. Cell 34 (2), 502–514. 10.1007/s13577-020-00458-z 33231844

[B125] Yang D. Qiao J. Wang G. Lan Y. Li G. Guo X. (2018). N6-Methyladenosine modification of lincrna 1281 is critically required for mesc differentiation potential. Nucleic Acids Res. 46 (8), 3906–3920. 10.1093/nar/gky130 29529255 PMC5934679

[B126] Yang L. Han B. Zhang Z. Wang S. Bai Y. Zhang Y. (2020a). Extracellular vesicle-mediated delivery of circular rna Scmh1 promotes functional recovery in rodent and nonhuman primate ischemic stroke models. Circulation 142 (6), 556–574. 10.1161/circulationaha.120.045765 32441115

[B127] Yang N. Ying P. Tian J. Wang X. Mei S. Zou D. (2020b). Genetic variants in M6a modification genes are associated with esophageal squamous-cell carcinoma in the Chinese population. Carcinogenesis 41 (6), 761–768. 10.1093/carcin/bgaa012 32047883

[B128] Yankova E. Blackaby W. Albertella M. Rak J. De Braekeleer E. Tsagkogeorga G. (2021). Small-molecule inhibition of Mettl3 as a strategy against myeloid leukaemia. Nature 593 (7860), 597–601. 10.1038/s41586-021-03536-w 33902106 PMC7613134

[B129] Yue Y. Zhang Q. Wu S. Wang S. Cui C. Yu M. (2020). Identification of key genes involved in jak/stat pathway in colorectal cancer. Mol. Immunol. 128, 287–297. 10.1016/j.molimm.2020.10.007 33248399

[B130] Zhang C. Samanta D. Lu H. Bullen J. W. Zhang H. Chen I. (2016). Hypoxia induces the breast cancer stem cell phenotype by hif-dependent and alkbh5-mediated m^6^a-demethylation of nanog mrna. Proc. Natl. Acad. Sci. U. S. A. 113 (14), E2047–E2056. 10.1073/pnas.1602883113 27001847 PMC4833258

[B131] Zhang L. Cai E. Xu Y. Liu Z. Zheng M. Sun Z. (2024a). Ythdf1 facilitates esophageal cancer progression via augmenting m6a-dependent Tinagl1 translation. Cell Signal 122, 111332. 10.1016/j.cellsig.2024.111332 39098703

[B132] Zhang P. Zhang W. Wang X. Li L. Lin Y. Wu N. (2024b). Bclaf1 drives esophageal squamous cell carcinoma progression through regulation of ythdf2-dependent Six1 mrna degradation. Cancer Lett. 591, 216874. 10.1016/j.canlet.2024.216874 38636894

[B133] Zhang S. Zhao B. S. Zhou A. Lin K. Zheng S. Lu Z. (2017). M(6)a demethylase Alkbh5 maintains tumorigenicity of glioblastoma stem-like cells by sustaining Foxm1 expression and cell proliferation program. Cancer Cell 31 (4), 591–606. 10.1016/j.ccell.2017.02.013 28344040 PMC5427719

[B134] Zhang X. Lu N. Wang L. Wang Y. Li M. Zhou Y. (2021). Recent advances of M(6)a methylation modification in esophageal squamous cell carcinoma. Cancer Cell Int. 21 (1), 421. 10.1186/s12935-021-02132-2 34376206 PMC8353866

[B135] Zhao F. Dong Z. Li Y. Liu S. Guo P. Zhang D. (2022). Comprehensive analysis of molecular clusters and prognostic signature based on m7g-related lncrnas in esophageal squamous cell carcinoma. Front. Oncol. 12, 893186. 10.3389/fonc.2022.893186 35912250 PMC9329704

[B136] Zhao R. Casson A. G. (2008). Epigenetic aberrations and targeted epigenetic therapy of esophageal cancer. Curr. Cancer Drug Targets 8 (6), 509–521. 10.2174/156800908785699306 18781897

[B137] Zhao W. Qi X. Liu L. Ma S. Liu J. Wu J. (2020). Epigenetic regulation of M(6)a modifications in human cancer. Mol. Ther. Nucleic Acids 19, 405–412. 10.1016/j.omtn.2019.11.022 31887551 PMC6938965

[B138] Zhao X. Yang Y. Sun B. F. Zhao Y. L. Yang Y. G. (2014). Fto and obesity: mechanisms of association. Curr. Diab Rep. 14 (5), 486. 10.1007/s11892-014-0486-0 24627050

[B139] Zhao Y. Li Y. Zhu R. Feng R. Cui H. Yu X. (2023). Rps15 interacted with Igf2bp1 to promote esophageal squamous cell carcinoma development via recognizing M(6)a modification. Signal Transduct. Target Ther. 8 (1), 224. 10.1038/s41392-023-01428-1 37264021 PMC10235050

[B140] Zheng F. Du F. Zhao J. Wang X. Si Y. Jin P. (2021). The emerging role of rna N6-methyladenosine methylation in breast cancer. Biomark. Res. 9 (1), 39. 10.1186/s40364-021-00295-8 34044876 PMC8161983

[B141] Zheng G. Dahl J. A. Niu Y. Fedorcsak P. Huang C. M. Li C. J. (2013). Alkbh5 is a mammalian rna demethylase that impacts rna metabolism and mouse fertility. Mol. Cell 49 (1), 18–29. 10.1016/j.molcel.2012.10.015 23177736 PMC3646334

[B142] Zheng Y. Liang G. Yuan D. Liu X. Ba Y. Qin Z. (2024). Perioperative toripalimab plus neoadjuvant chemotherapy might improve outcomes in resectable esophageal cancer: an interim analysis of a phase iii randomized clinical trial. Cancer Commun. (Lond) 44 (10), 1214–1227. 10.1002/cac2.12604 39221992 PMC11483553

[B143] Zheng Z. Lin F. Zhao B. Chen G. Wei C. Chen X. (2025). Alkbh5 suppresses gastric cancer tumorigenesis and metastasis by inhibiting the translation of uncapped Wrap53 rna isoforms in an m6a-dependent manner. Mol. Cancer 24 (1), 19. 10.1186/s12943-024-02223-4 39815301 PMC11734446

[B144] Zhou C. Molinie B. Daneshvar K. Pondick J. V. Wang J. Van Wittenberghe N. (2017). Genome-wide maps of M6a circrnas identify widespread and cell-type-specific methylation patterns that are distinct from mrnas. Cell Rep. 20 (9), 2262–2276. 10.1016/j.celrep.2017.08.027 28854373 PMC5705222

[B145] Zhou K. I. Shi H. Lyu R. Wylder A. C. Matuszek Ż. Pan J. N. (2019). Regulation of Co-transcriptional pre-mrna splicing by M(6)a through the low-complexity protein hnrnpg. Mol. Cell 76 (1), 70–81. 10.1016/j.molcel.2019.07.005 31445886 PMC6778029

[B146] Zhou Y. Xue X. Luo J. Li P. Xiao Z. Zhang W. (2023). Circular rna circ-firre interacts with hnrnpc to promote esophageal squamous cell carcinoma progression by stabilizing Gli2 mrna. Cancer Sci. 114 (9), 3608–3622. 10.1111/cas.15899 37417427 PMC10475760

[B147] Zhu T. Roundtree I. A. Wang P. Wang X. Wang L. Sun C. (2014). Crystal structure of the yth domain of Ythdf2 reveals mechanism for recognition of N6-methyladenosine. Cell Res. 24 (12), 1493–1496. 10.1038/cr.2014.152 25412661 PMC4260350

[B148] Zou J. Ma Q. Gao C. Yang M. Wen J. Xu L. (2024). Wtap promotes proliferation of esophageal squamous cell carcinoma via M(6)a-dependent epigenetic promoting of Ptp4a1. Cancer Sci. 115 (7), 2254–2268. 10.1111/cas.15924 38746998 PMC11247548

